# The effect of Zuogui-Jiangtang-Yishen decoction on the intestinal flora’s response to L-α-phosphatidylcholine and L-tyrosine in patients with diabetic kidney disease: an *in vitro* study

**DOI:** 10.3389/fphar.2025.1573514

**Published:** 2025-06-11

**Authors:** Yeshi Yin, Changhui Zhao, Qin Xiang, Zongyan Li, Xiu Liu, Changhui Hu, Rong Yu

**Affiliations:** ^1^ Guangxi Key Laboratory of Animal Reproduction, Breeding and Disease Control, Guangxi Zhuang Autonomous Region Engineering Research Center of Veterinary Biologics, College of Animal Science and Technology, Guangxi University, Nanning, China; ^2^ Key Laboratory of Comprehensive Utilization of Advantage Plants Resources in Hunan South, College of Chemistry and Bioengineering, Hunan University of Science and Engineering, Yongzhou, China; ^3^ School of Traditional Chinese Medicine, Hunan University of Chinese Medicine, Changsha, China; ^4^ Depatment of Clinical Laboratory, The Fourth People’s Hospital of Yongzhou, Yongzhou, China

**Keywords:** Zuogui-Jiangtang-Yishen decoction, diabetic kidney disease, intestinal flora, L-α-phosphatidylcholine, L-tyrosine, *in vitro* fermentation

## Abstract

**Background:**

Animal and cell studies have demonstrated that Zuogui-Jiangtang-Yishen decoction (ZGJTYS) has a favorable effect on the treatment of diabetic kidney disease (DKD). Our previous clinical research also showed that ZGJTYS prevents DKD in a manner similar to that of benazepril. Nevertheless, the interactions between ZGJTYS and the human gut microbiota require further investigation, particularly its interference in the intestinal flora response to food ingredients that may increase DKD risk, such as L-α-phosphatidylcholine and L-tyrosine.

**Objective:**

The aim of this study was to evaluate the regulatory function of ZGJTYS on human gut microbiota and explore the effect of ZGJTYS on the intestinal flora response to L-α-phosphatidylcholine and L-tyrosine.

**Methods:**

ZGJTYS was prescribed from the First Affiliated Hospital of Hunan University of Chinese Medicine. High-throughput sequencing of bacterial 16S RNA genes and fungal internal transcribed spacer (ITS) sequences was used for intestinal flora analysis. An *in vitro* gut microbiota simulation model was used to investigate the effect of ZGJTYS on the intestinal flora’s response to L-α-phosphatidylcholine and L-tyrosine. Ultra-high-performance liquid chromatography and mass spectrometry were used for non-targeted metabolomics analysis.

**Results:**

Compared to the control group, the microbial diversity of DKD was significantly reduced by ZGJTYS treatment; three bacterial genera, including *Parabacterioids*, were significantly higher; eight bacterial genera, including *Prevotella_9*, and the linoleic acid content were significantly lower. A receiver operating characteristic curve analysis using *Parabacterioids* and *Prevotella_9* showed an area under the curve greater than 0.75, indicating good predictive performance. ZGJTYS intervention restored some of the normal bacterial genera, such as *Rickettsia* and *Metarhizium*, which were regulated by L-α-phosphatidylcholine and L-tyrosine. Furthermore, ZGJTYS effectively restored several significantly different Kyoto Encyclopedia of Genes and Genomes metabolic pathways related to immunity and disease to normal, such as efferocytosis and tryptophan metabolism.

**Conclusion:**

ZGJTYS was found to effectively restore the microbiota that were altered by L-α-phosphatidylcholine and L-tyrosine to normal, along with their metabolites. However, the mechanism by which ZGJTYS exerts its preventive and therapeutic effects on DKD through the gut microbiota still requires further study.

## 1 Introduction

The value of the early detection and prevention of diabetic kidney disease (DKD) cannot be overstated. DKD affects 30–40% of patients with diabetes mellitus (DM), and it is one of the most prevalent and severe DM complications. In addition, it is the leading cause of end-stage renal disease (ESRD) (Alicic et al., 2017). Approximately 537 million adults (20–79 years old) worldwide had diabetes in 2021. By 2045, that figure is expected to rise to 783 million (Kumar et al., 2024), its prevalence rate is still alarmingly escalating across every age group, ranging from children to older adults (Collaborators, 2023; Sun et al., 2022). Moreover, current DKD treatment options are limited, and their therapeutic effects are often unsatisfactory (Wang and Zhang, 2024). Even with tight glycemic control and the maintenance of glycosylated hemoglobin values lower than 7% have produced little relief (Rodriguez-Gutierrez and Montori, 2016). The high incidence and low remission rate of DKD has resulted in an enormous economic burden worldwide (Mcgrath and Edi, 2019; Ruiz-Ortega et al., 2020).

Although, many factors are included in DKD progression, the pathogenesis of DKD development remains unclear (Bhupesh et al., 2023). Recently, an increasingly emphasized correlation between DKD and intestinal dysbiosis was reported, and the theory of the “gut–kidney axis” was proposed to better understand DKD (Huang et al., 2024). A meta-analysis showed that increasing the *Butyricicoccus*, *Faecalibacterium*, *Lachnospira,* and Roseburia genera, and depleting the proportion of the *Hungatella*, *Escherichia, Citrobacter*, and *Klebsiella* genera has good effects in treating in DKD (Han et al., 2022; Wang et al., 2022a). In addition, 10 key operational taxonomic units (OTUs), such as *Lactobacillus* and *Lachnospiraceae*, have been selected as biomarkers for DKD diagnosis (Shang et al., 2022). Additionally, intestinal microbial metabolites, including short-chain fatty acids (SCFAs), bile acids, trimethylamine-N-oxide (TMAO), indoxyl sulfate (IS), and p-cresol sulfate (p-CS), are closely related to DKD occurrence and development (Huang et al., 2024). Moreover, nutritional intervention and microecological intervention that target intestinal flora have achieved good results in DKD treatment (Ghosh et al., 2024; Peride et al., 2024).

The DKD pathogenesis mechanisms are complex, and the use of hypoglycemic drug treatments, such as SGLT2 inhibitors, GLP-1R agonists, and antihypertensive drugs, are often not ideal (Patel et al., 2020; Wang and Zhang, 2024). Botanical drugs may have advantages in DKD treatment because it contains a variety of effective ingredients. Some clinical studies have also demonstrated the significant efficacy of Tangshen formula, Shenqi Dihuang decoction, Buyang Huanwu decoction, and the Zicuiyin decoction in treating DKD (Shen et al., 2024). However, the mechanisms behind these protective effects remain poorly understood (Deng et al., 2024).

Our previous studies demonstrated that ZGJTYS has beneficial effects in DKD treatment (Yi et al., 2023; Chen et al., 2015; Xia et al., 2011). Although, our previous studies showed that ZGJTYS can regulate many signal pathways, such as miRNA-27a/Wnt/β-catenin (Hu et al., 2024) and TMAO-mROS-NLRP3 axis activated pyroptosis (Yi et al., 2023), we speculated that it might also alleviate DKD through regulated gut microbiota. Therefore, in this study, we assessed the regulatory function of ZGJTYS on human gut microbiota and its metabolic activation of L-α-phosphatidylcholine and L-tyrosine using an *in vitro* model.

## 2 Materials and methods

### 2.1 Ethical approval

All procedures performed in studies involving human volunteers were in accordance with the ethical standards of the 1964 Helsinki Declaration (and its latest amendments) or comparable ethical standards. The procedures were approved by the research committees of the First Affiliated Hospital of Hunan University of Chinese Medicine (HN-LL-KY-2021-035-01) and the Hunan University of Science and Engineering (HUSE-LL-20210,310).

### 2.2 ZGJTYS preparation and component analysis

ZGJTYS formula consists of nine medicinal herbs; the specific details are provided in Table 1. All traditional Chinese medicines were sourced from The First Affiliated Hospital of Hunan University of Chinese Medicine and prepared according to The ConPhyMP Guidelines (Heinrich et al., 2022). In brief, each herb was soaked in 10 times its volume of water for 30 min, boiled over high heat, and then simmered for 1.5 h. The decoction was filtered through gauze, and the residue was reboiled with 8 times its volume of water for 1 h. The combined filtrates were concentrated to 300 mL of fluid extract, lyophilized under reduced pressure to obtain a dry powder, and stored at 4°C.

**TABLE 1:** Specific information about ZGJTYS.

Latin scientific name	Dosage (g)	Batch number
*Astragalus mongholicus* Bunge	18	CK21063002
*Salvia miltiorrhiza* Bunge	9	HY21061501
*Dioscorea opponita* Thunb	12	CK21061807
*Coptis chinensis* Franch	6	HH21060704
*Achyranthis Bidentatae* Bl	9	SL21060301
*Leonurus japonicus* Houtt	9	200,901
*Rehmannia glutinosa* Libosch	12	2,103,033
*Cornus officinalis* Sieb. Et Zucc	12	TH21060703
*Zea mays* L	12	2,021,042,602

Approximately 0.5 g of the sample was homogenized with 10 mL of absolute methanol. After ultrasonication and centrifugation, the supernatant was collected and filtered through a 0.22 μm membrane filter for analysis. Chromatographic separation of ZGJTYS was performed using an Agilent 1200 HPLC system (Agilent Technologies, United States) equipped with a Welch Ultimate AQ-C18 column (4.6 mm × 250 mm, 5 μm). The column temperature was maintained at 25°C, with an injection volume of 10 μL, a flow rate of 0.8 mL/min, and a detection wavelength of 254 nm. The mobile phase consisted of eluent B (methanol) and eluent A (0.1% aqueous formic acid). Detailed analytical procedures and standard information were based on previous research conducted by our group (Yi et al., 2023).

### 2.3 Chemicals and reagents

L-α-phosphatidylcholine and L-tyrosine were ordered from Sigma-Aldrich^®^ (Saint Louis, United States). Chromatographic grade acetic acid, propionic acid, butyric acid, isobutyric acid, valeric acid, and isovaleric acid were purchased from the Aladdin Biochemical Technology Co., Ltd. (Shanghai, China). Peptone, tryptone, and yeast extract were purchased from Oxoid Ltd. (Basingstoke, United Kingdom). Liquid chromatography–mass spectrometry (LC-MS) grade methanol (MeOH) was purchased from Fisher Scientific (Loughborough, United Kingdom). 2-Amino-3-(2-chloro-phenyl)-propionic acid was obtained from Aladdin (Shanghai, China). Ultrapure water was generated using a Milli-Q system (Millipore, Bedford, United States). Starch, ZnSO_4_, and other chemical agents were purchased from the Sangon Biotech Co., Ltd. (Shanghai, China).

### 2.4 Fecal sample collection

A total of 12 health volunteers (six males and six females) with an average age of 22 years were recruited from the Hunan University of Science and Engineering (the HF group). All volunteers had no history of smoking or drinking, and none had used antibiotics in the 3 months prior to sampling. A total of 12 DKD patients (six males and six females) with an average age of 60 years were recruited from the First Affiliated Hospital of Hunan University of Chinese Medicine (the CF group). Among these DKD patients, only three had a history of smoking and drinking, and no dietary control was implemented. All patients had not used antibiotics prior to sampling but were already taking hypoglycemic drugs. A total of 12 non-metabolic disease patients (six males and six females) with an average age of 59.5 years were recruited from The Fourth People’s Hospital of Yongzhou (the YF group). Fecal samples were collected and preserved in a deoxygenated glycerol buffer within 2 h after defecation (Zhang et al., 2022), then stored at −80°C.

### 2.5 ZGJTYS treatment with simulated upper gastrointestinal fluid

The ZGJTYS was pretreated before being used for *in vitro* fermentation using simulated saliva, gastric juice, and small intestinal fluid in accordance with the previous research (Minekus et al., 2014). In brief, the *in vitro* digestion protocol commenced by suspending 5 g of ZGJTYS in 3.5 mL of simulated salivary fluid (SSF) electrolyte stock solution, followed by the sequential addition of 0.5 mL human salivary α-amylase (Type IX-A, 1,500 U/mL final concentration; Sigma-Aldrich), 25 μL of 0.3 M CaCl_2_, and 975 μL ultrapure water under vortex mixing. After preheating at 37°C with agitation at 120 rpm for 2 min, the oral phase digestate was transitioned to gastric simulation by adding 7.5 mL simulated gastric fluid (SGF) electrolyte stock solution, 1.6 mL porcine pepsin (25,000 U/mL; Sigma-Aldrich), 5 μL 0.3 M CaCl_2_, and 695 μL ultrapure water, with pH adjusted to 3.0 using 0.2 mL 1 M HCl under real-time monitoring. This gastric mixture was incubated at 37°C with orbital shaking at 120 rpm for 2 h before proceeding to the intestinal phase, where 20 mL of gastric chyme was blended with 11 mL simulated intestinal fluid (SIF) electrolyte stock solution, 5.0 mL porcine trypsin (800 U/mL; Sigma-Aldrich), 2.5 mL 160 mM bile salts, 40 μL 0.3 M CaCl_2_, and 1.31 mL ultrapure water. The pH was then neutralizated to 7.0 with 0.15 mL 1 M NaOH. The final intestinal digestion was carried out at 37°C for 2 h with mechanical mixing, followed by rapid ice-cooling and storage at −80°C for subsequent analyses. The SSF, SGF, and SIF electrolyte stock solutions were prepared according to the formula described previously (Minekus et al., 2014).

### 2.6 *In vitro* fermentation of ZGJTYS with gut microbiota from health volunteers and DKD patients

The culture medium of VI was prepared according to our previous study (Hu et al., 2019). First, the following components were mixed and the pH was adjused to 6.5 using 1 M HCl before autoclaving: 3 g/L peptone, 3 g/L tryptone, 4.5 g/L yeast extract, 0.5 g/L mucin, 0.4 g/L bile salts No. 3, 4.5 g/L NaCl, 2.5 g/L KCl, 4.5 g/L MgCl2·6H2O, 1 mL Tween 80, 0.2 g/L CaCl2.6H2O, 0.4 g/L KH2PO4, 3.0 g/L MgSO4·7H2O, 0.32 g/L MnCl2·4H2O, 0.1 g/L FeSO4·7H2O, 0.18 g/L CoSO4·7H2O, 0.1 g/L CaCl2·2H2O, 0.18 g/L ZnSO4·7H2O, 0.01 g/L CuSO4·5H2O, and 0.092 g/L NiCl2·6H2O. Second, after autoclaving and cooling, L-cysteine hydrochloride and hemin were added to final concentrations of 0.8 g/L and 0.05 g/L, respectively. The concentration of ZGJTYS used in this study, following treatment with simulated upper gastrointestinal fluid, was 15 g/L, which was calculated according to our dosage prescribed clinically for patients (25 g/day/person). Approximately 20% of administered drugs reach the colon, and the volume of the colon is ∼0.3 L. The concentration used for L-α-phosphatidylcholine and L-tyrosine was 2 g/L, which was calculated based on the published literature (Kikuchi et al., 2019; Wang et al., 2011). The ZGJTYS and L-α-phosphatidylcholine (dissolved in 50% ethanol, 100 mg/mL) was added into the autoclaved VI media after filtering through 0.2-μm filter, and the L-tyrosine was autoclaved with the VI medium. A 10% fecal slurry was prepared and then filtered through four layers of gauze. A 500 μL fecal slurry was then added to the different prepared fermentation tubes (contained 9.5 mL culture medium) in an anaerobic chamber (10% H_2_, 10% CO_2_, and 80% N_2_) and then incubated at 37°C. Samples were collected at 48 h for further analyses.

### 2.7 16S rRNA gene and internal transcribed spacer (ITS) analysis of the gut microbiota

The V3–V4 region of the bacterial 16S rRNA genes was amplified using the primers 338F (5′-ACT CCT ACG GGA GGC AGC AG-3′) and 806R (5′-GGA CTA CHV GGG TWT CTA AT-3′) (Dennis et al., 2013). The ITS2 region was amplified using ITS3 (5′-TCG TCG GCA GCG TCA GAT GTG TAT AAG AGA CAG GCA TCG ATG AAG AAC GCA GC -3′) and ITS4 (5′- GTC TCG TGG GCT CGG AGA TGT GTA TAA GAG ACA GTC CTC CGC TTA TTG ATA TGC -3′) (Borruso et al., 2021). The amplicons were then sequenced on the Illumina MiSeq 300 PE platform by the Mingke Biotechnology (Hangzhou) Co., Ltd. (Hangzhou, China). The resulting reads were analyzed using the Quantitative Insights Into Microbial Ecology (QIIME 2) pipeline. High-quality sequences were taxonomically classified in defining the OTUs using Mothur at a 97% sequence similarity (Schloss et al., 2009). The representative sequences were chosen and classified using the Ribosomal Database Project (RDP) classifier method (Cole et al., 2009; Wang et al., 2007) and aligned against the SILVA database (https://www.arb-silva.de/documentation/release-123/). High-throughput sequencing data of the bacteria and fungi were analyzed using the cloud platform tools (http://www.cloud.biomicroclass.com/CloudPlatform and http://cloud.mingkebio.com/#/latest-tools).

### 2.8 Non-targeted metabolomics analysis

A non-targeted metabolomics analysis was conducted by the Suzhou PANOMIX Biomedical Tech Co., Ltd. (Suzhou, China). The LC analysis was then performed on a Vanquish Ultra-High-Performance Liquid Chromatography (UHPLC) System (Thermo Fisher Scientific, United States) according to previously published methods (Zelena et al., 2009), and an ACQUITY UPLC^®^ HSS T3 column (150 × 2.1 mm, 1.8 µm) was used (Waters, Milford, MA, United States). Mass spectrometric detection of metabolites was performed on a Q Exactive Focus (Thermo Fisher Scientific, United States) with an electrospray ionization (ESI) ion source according to the published methods (Want et al., 2013). The non-targeted metabolomics data were analyzed using cloud tools (https://www.biodeep.cn/home).

### 2.9 Statistical analysis

SPSS software (version 27.0; SPSS Inc., Chicago, IL, United States) was selected for the statistical analyses in this study. Plot cladograms and significantly different bacterial taxa were analyzed using LEfSe software, which is included in the cloud tools (http://www.cloud.biomicroclass.com/CloudPlatform). A P-value less than 0.05 was considered to indicate statistically significantly difference.

## 3 Results

### 3.1 Basic data obtained for the analysis

A total of 252 samples were subjected to high-throughput sequencing of bacterial 16S rDNA genes and the fungal ITS region (Supplementary Table S1). After flattening, 43,960 reads per sample were utilized for the bacterial diversity analysis. A total of 3,109 OTUs were identified, distributed in 655 bacterial genera. A total of 29,865 reads per sample were used for the fungal diversity analysis. In addition, a total of 4,174 OTUs were identified that were distributed in 1,279 fungal genera. A non-target metabolomics analysis was conducted on 108 *in vitro* cultured samples, and a total of 650 compounds were identified.

### 3.2 Analysis of the bacterial and fungal diversity in the original fecal samples

The high-throughput sequencing results indicated that the top five bacterial genera in these fecal samples were *Bacteroides*, *Prevotella*_9, *Blautia*, *Bifidobacterium*, and *Megamonas* (Supplementary Figure S1A). The top five fungal genera were *Metarhizium*, *Geotrichum*, *Aspergillus*, *Kazachstania*, and *Diutina* (Supplementary Figure S1B). The bacterial alpha diversity analysis showed that the ACE, Chao1, and Observed_species were significantly lower in the CF group than in the HF group, but there was no significant difference between the HF and YF groups (Supplementary Table S2). The fungal alpha diversity analysis demonstrated that there were no significant differences in the ACE, Chao1, Observed_species, Shannon, and Simpson indexes among the HF, CF, and YF groups (Supplementary Table S2).

**FIGURE 1:**
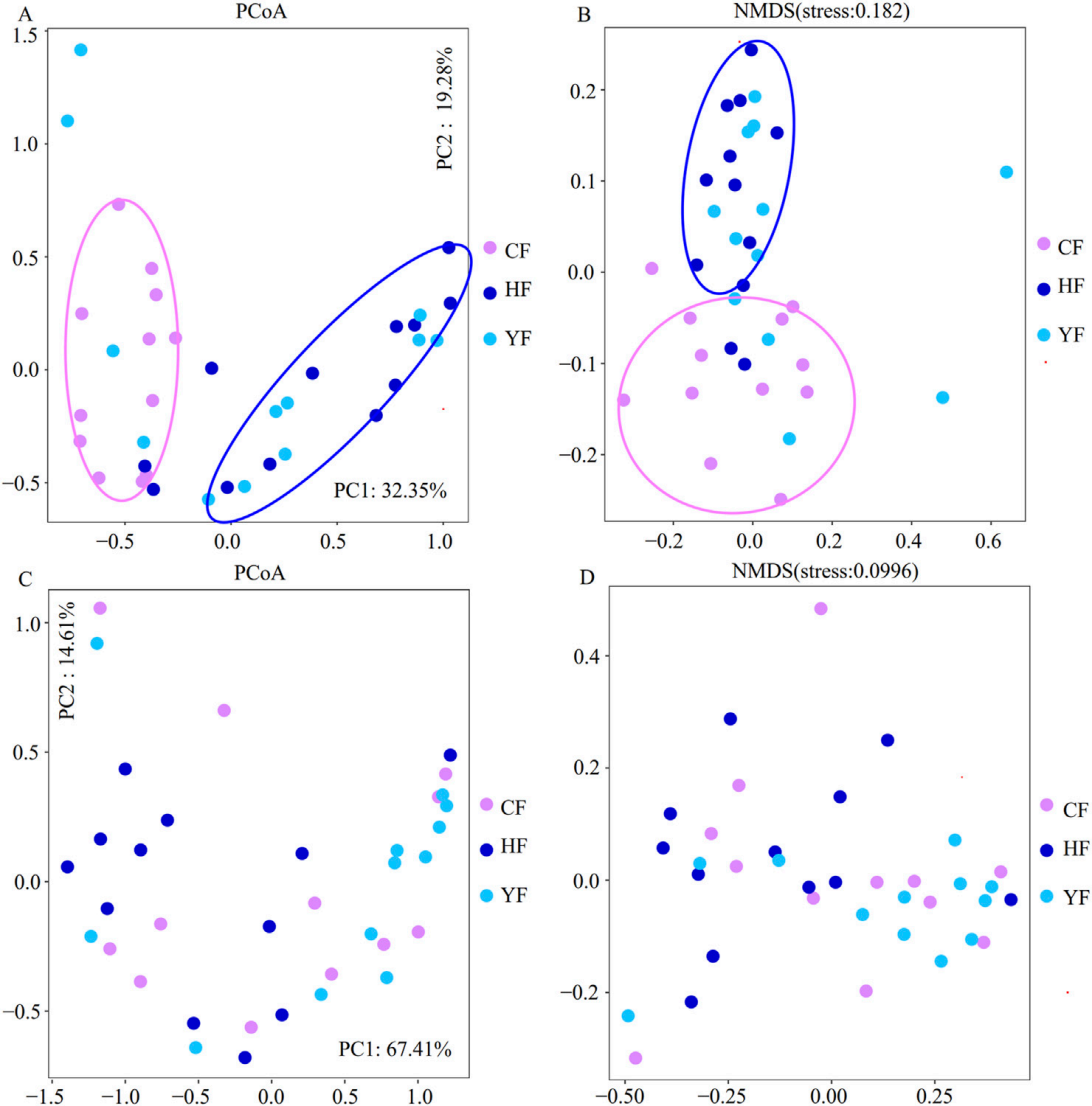
Analysis of the bacterial and fungal beta diversity in the original fecal samples. **(A)** and **(B)** are the bacterial beta diversity in the original fecal samples; **(C)** and **(D)** are the fungal beta diversity in the original fecal samples. CF represents the DKD group, HF represents the healthy college student volunteer control group, and YF represents the middle-aged and elderly non-metabolic disease control group.

Beta diversity analysis results indicated that both the principal component analysis (PCoA) and the nonmetric multidimensional scaling (NMDS) clustered the bacterial compositions of the HF and YF fecal samples closely together, indicating high similarity as they clustered within the same branch. However, both analyses showed significant differences compared to the CF group (Figures 1A,B). For the fungal composition analysis, there was no satisfactory clustering among these groups according to their sources (Figures 1C,D). This indicated that the differences in the bacterial community among these groups were more pronounced.

**FIGURE 2:**
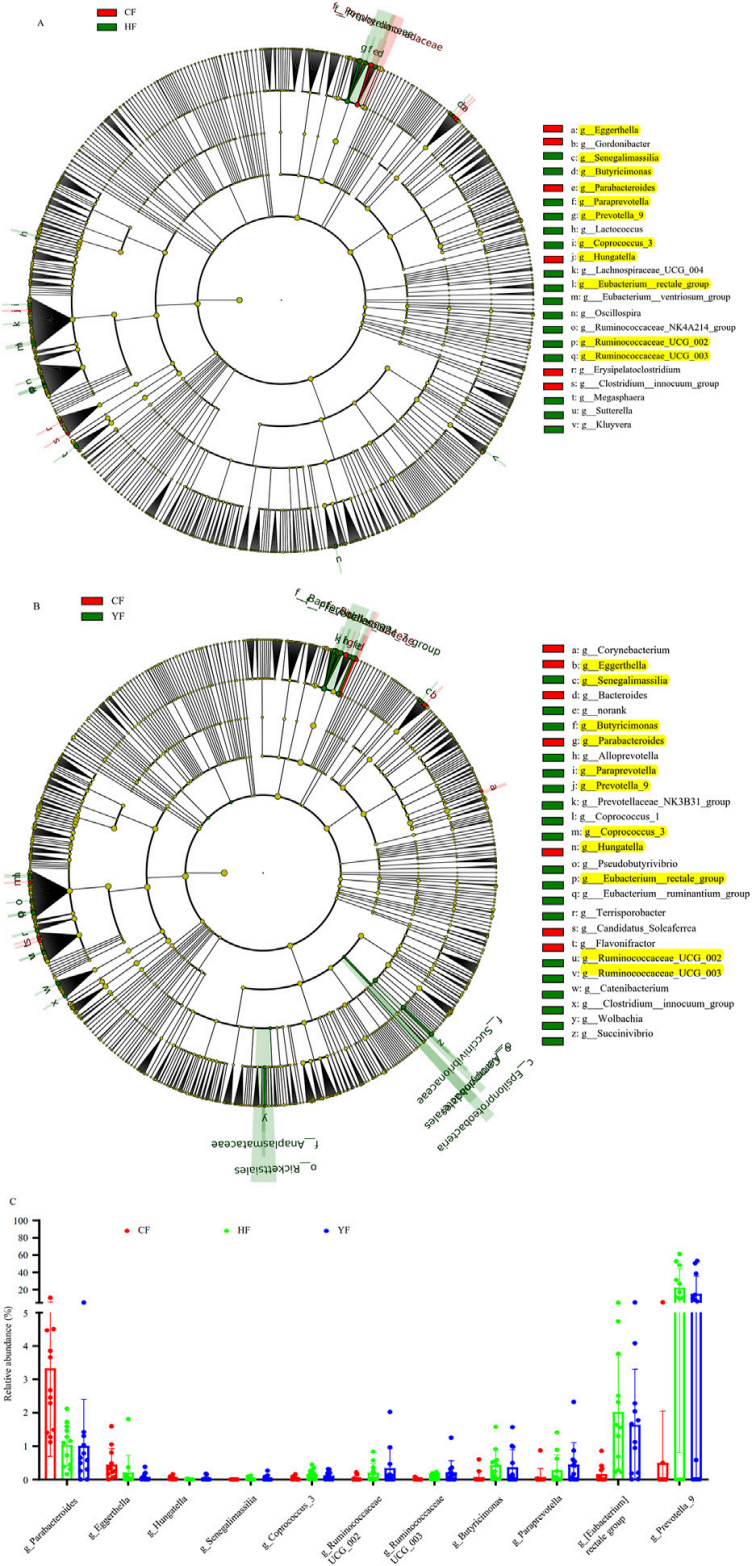
Lefse analysis of the bacterial composition differences in the original fecal samples. **(A)** represents the significantly different bacterial genera between the CF and HF groups, **(B)** represents the significantly different bacterial genera between the CF and YF groups, and **(C)** represents the relative abundance analysis of bacterial genera that showed significant differences in both **(A,B)**. The yellow marked bacterial genera indicate significant differences in both **(A,B)**. CF represents the DKD group, HF represents the healthy college student volunteer control group, and YF represents the middle-aged and elderly non-metabolic disease control group.

**FIGURE 3:**
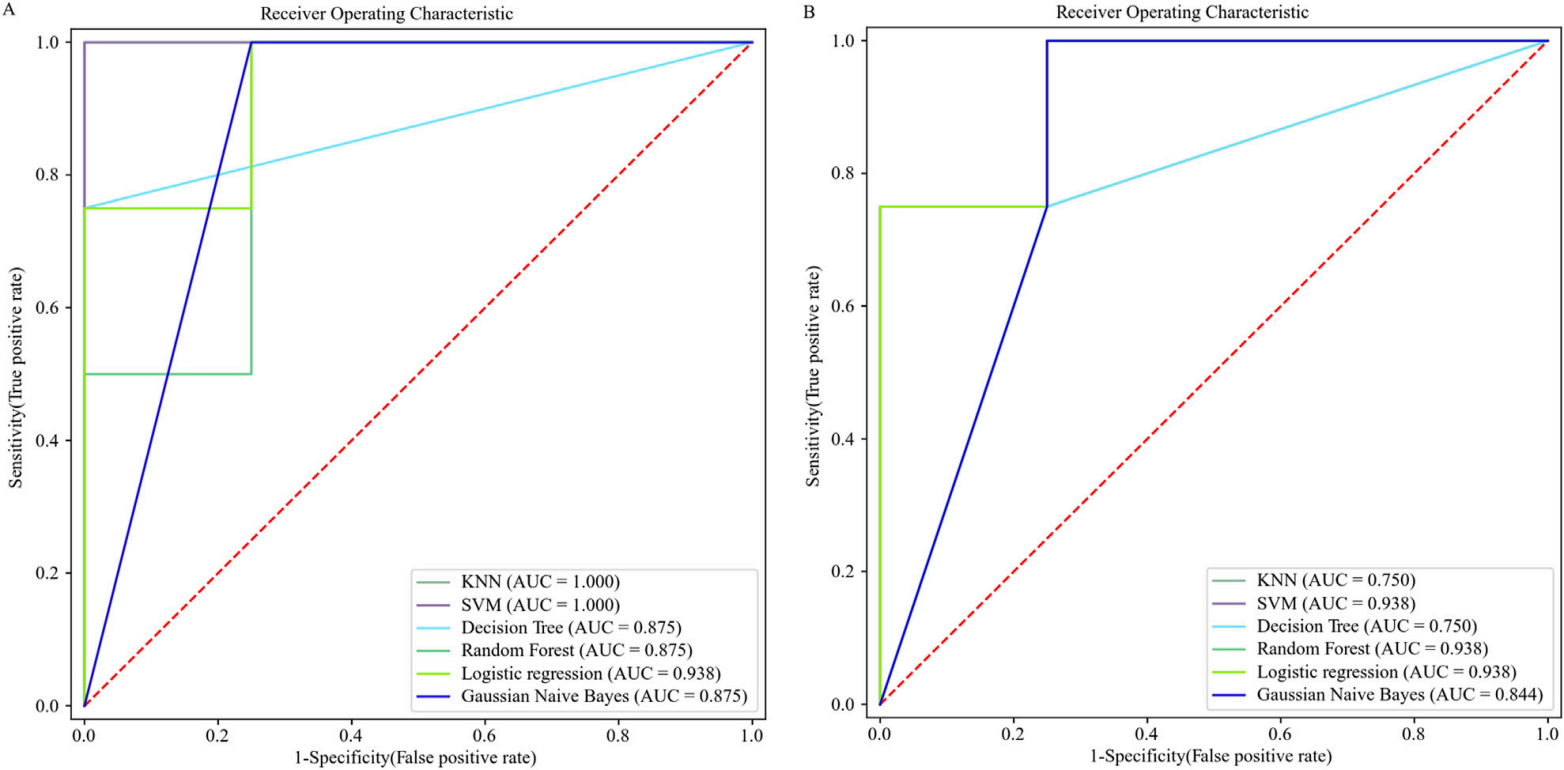
ROC diagnostic curves based on differential bacterial genera. **(A)**. ROC diagnostic curve generated using 11 differential bacterial genera. **(B)**. ROC diagnostic curve generated using *Parabacterioids* and *Prevotella_9*.

The LEfSe analysis shows that there were six and 16 bacterial genera in the CF group that were significantly higher and lower than those in the HF group, respectively (Figure 2A), and there were seven and 19 bacterial genera in the CF group that were significantly higher and lower than those in the YF group, respectively (Figure 2B). A further comparative analysis of the significantly different genera showed that the bacterial genera of *Parabacterioids*, *Eggerthella*, and *Hungatella* were significantly higher in the CF group than in the HF and YF groups. However, the bacterial genera of *Senegalimassilia*, *Coprococcus*_3, *Ruminococcae*_UCG_002, *Ruminococcae*_UCG_003, *Butyricimonas*, *ParaPrevotella*, *Eubacterium_rectale*_group, and *Prevotella*_9 were significantly lower in the CF group than in the HF and YF groups (Figure 2C). We then performed receiver operating characteristic (ROC) diagnostic curve analysis using these 11 significantly different bacterial genera, obtaining an area under the curve (AUC) exceeding 0.875 (Figure 3A). Due to the relative abundance of *Parabacterioids* exceeding 3% in the CF group and the relative abundance of *Prevotella 9* exceeding 20% in the HF group, we conducted a separate ROC analysis using these two highly abundant differential genera, which yielded a baseline AUC exceeding 0.75 (Figure 3B). In the fungal LEfSe analysis, the relative abundance of *Trichosporon*, *Piromyces*, *Tahromyces*, and *Mucor* in the CF group was significantly lower than that in the HF group, while the relative abundance of *Chaetomium*, *Phaeosphaeria*, and *Cadophora* was significantly higher in the YF group (Supplementary Figure S2).

Although the YF group had similar ages as the CF group (DKD patients), other non-metabolic diseases present in the YF volunteers could have affected the analysis. Moreover, the microbiota structures were very similar between the healthy control group (HF group) and the YF group. To simplify the analysis and present the results more intuitively, the YF group data were excluded in the subsequent analysis.

### 3.3 Regulatory effects of ZGJTYS on the human gut microbiota *in vitro*

Supplementary Figure S3 shows that the VI medium has a good simulation effect on both the HF and CF fecal bacteria and fungi communities *in vitro*. Although a better simulation effect was found for HF samples, the CF samples were significantly different from the HF samples, which were primarily distributed above the X-axis. Moreover, the major bacterial genera showed significant differences between CF-VI and HF-VI after the *in vitro* simulated culture. The relative abundance of *Parabacterioids* was significantly higher in the CF_VI group than in the HF_VI group, and the relative abundance of *Prevotella_9* was significantly higher in the HF_VI group than in the CF_VI group (Supplementary Figure S4). These results suggested that simulated fermentation can be used for the *in vitro* study on the effects of ZGJTYS, L-α-phosphatidylcholine, and L-tyrosine regulation on fecal microbiota.

Non-target metabolomics were used to detect the metabolic effects of artificial upper gastrointestinal simulation fluid on ZGJTYS prior to investigating the regulatory effect of drugs on the gut microbiota. As a result, a total of 492 compounds were identified between the original drug and the drug treated with artificial upper gastrointestinal simulation fluid. Among these, 139 compounds were significantly upregulated and 31 compounds were significantly downregulated. Beta-alanine metabolism; alanine, aspartate, and glutamate metabolism; phenylpropanoid biosynthesis; tryptophan metabolism; phenyalanine, tyrosine, and tryptophan biosynthesis; and lysine degradation were significantly enriched in the protein metabolism related signaling pathways between these two groups (Supplementary Figure S5). Considering the changes after upper gastrointestinal simulation fluid treatment, ZGJTYS treatment was used for subsequent studies.

**FIGURE 4:**
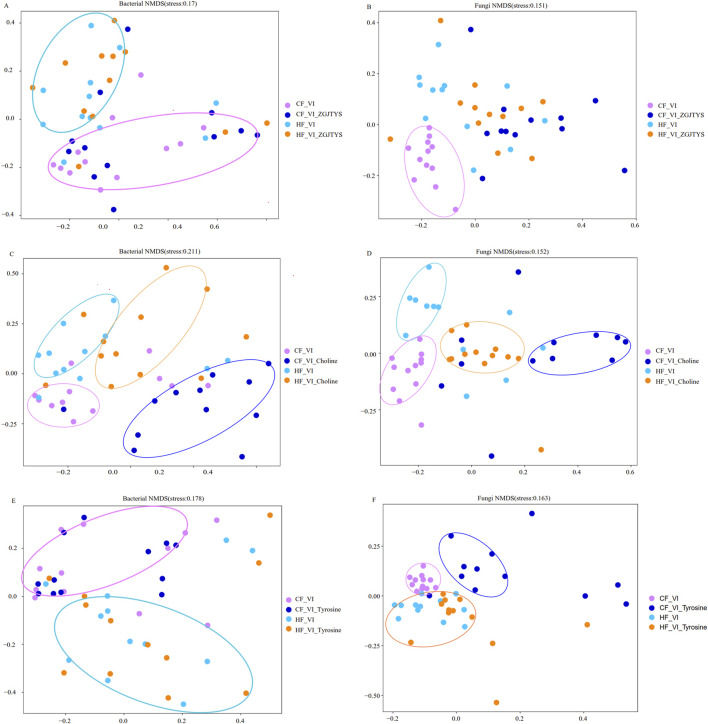
Beta diversity analysis of the effects of ZGJTYS, L-α-phosphatidylcholine, and L-tyrosine on the compositions of the bacterial and fungal communities. **(A,C,E)** are the results of the bacterial beta diversity analysis, while **(B,D,F)** are the results of the fungal beta diversity analysis. CF represents the DKD group, and HF represents the healthy college student volunteer control.

In the analysis of the intestinal flora response to ZGZTYS, the bacterial and fungal alpha diversity results indicated that no significant effects were found in the HF_VI samples. The bacterial and fungal diversity indices ACE, Chao1, Observed_species, Shannon, and Simpson did not show significant differences between the HF_VI and HF_VI_ZGZTYS groups (Supplementary Table S2). Although the bacterial diversity did not significantly change after ZGZTYS intervention in the CF_VI group, the fungal diversity significantly changed after ZGZTYS intervention (Supplementary Table S2). The ACE, Chao1, Observed_species, Shannon, and Simpson fungal diversity indexes were significantly lower in the CF_VI_ZGJTYS group than in the CF_VI group (Supplementary Table S2). A further beta diversity analysis showed that the ZGZTYS intervention had a slight effect on the microbial composition of the HF_VI and CF_VI groups, with the HF_VI group clustered together with the HF_VI_ZGJTYS group and the CF_VI_ZGJTYS group clustered together with CF_VI (Figure 4A). However, the ZGJTYS intervention had a significant impact on the fungal composition of the CF_VI group, and the CF_VI_ZGJTYS group was significantly distant from the CF_VI group and closer to the relative clustering relationship of the HF_VI and HF_VI_ZGJTYS groups (Figure 4B). Moreover, the LEfSe analysis also showed that the ZGJTYS intervention had little effect on bacteria. No significant differences in the bacterial genera were detected between the HF_VI and HF_VI_ZGJTYS groups (Supplementary Figure S6A), and only eight bacterial genera showed significant differences between the CF_VI and CF_VI_ZGJTYS groups (Supplementary Figure S6B). However, ZGJTYS had a greater impact on fungal communities. There were 14 fungal genera with significant differences between the HF_VI and HF_VI_ZGJTYS groups (Supplementary Figure S6C), and 18 fungal genera with significant differences between the CF_VI and CF_VI_ZGJTYS groups (Supplementary Figure S6D).

### 3.4 Regulatory effect of ZGJTYS on the intestinal flora response to L-α-phosphatidylcholine

In the analysis of the interventional functions of L-α-phosphatidylcholine, the bacterial and fungal alpha diversity results revealed that significant changes occurred in both the HF_VI and the CF_VI groups (Supplementary Table S2). Significant differences were detected in the ACE and Observed_species of bacteria and fungi between the HF_VI and HF_VI_Choline groups (Supplementary Table S2). However, L-α-phosphatidylcholine had a greater impact on fungi than on bacteria in the CF_VI group. There were significant differences in the ACE, Chao1, Shannon, Observed_species, Shannon, and Simpson fungal diversity indexes between the CF_VI and CF_VI_Choline groups (Supplementary Table S2), while only the Observed_species showed significant differences in the bacterial diversity indexes between the CF_VI and CF_VI_Choline groups (Supplementary Table S2). The beta diversity analysis showed that the L-α-phosphatidylcholine had a significant impact on the bacterial and fungal composition on the HF_VI and CF_VI groups. Both the HF_VI group and the CF_VI groups exhibited an overall rightward shift in their bacterial and fungal clustering circles after L-α-phosphatidylcholine intervention (Figures 4C,D). The Lefse analysis also discovered that the L-α-phosphatidylcholine intervention had a significant impact on both bacteria and fungi. A total of 41 significantly different bacterial genera were detected between the HF_VI and HF_VI_Choline groups (Supplementary Table S3), with the abundance of 30 bacterial genera in the HF_VI_Choline group (25 bacterial genera appeared simultaneously with choline in the PubMed paper) significantly higher than in the HF_VI group (Supplementary Tables S2, S3). A total of 68 significantly different bacterial genera were detected between the CF_VI and CF_VI_Choline groups (Supplementary Table S3), with the abundance of 54 bacterial genera in the CF_VI_Choline group (including 42 bacterial genera that appeared simultaneously with choline in the PubMed paper) significantly higher than in the CF_VI group (Supplementary Tables S2, S3). There were 13 bacterial genera *Achromobacter*, *Aliihoeflea*, *Alistipes*, *Bacteroides*, *Comamonas*, *Halomonas*, *Microcella*, *Parabacteroides*, *Pelagic bacteria*, *Pseudomonas*, *Ralstonia*, *Serratia*, and *Sphingobacteria* were both significantly enhanced in the HF_VI_Choline and CF_VI_Choline groups after the L-α-phosphatidylcholine addition; five bacterial genera belonging to *Actinomyces*, *Erysipelatoclostridium*, *Gemella*, *Granulicatella*, and *Streptococcus* were both significantly reduced after the L-α-phosphatidylcholine treatment. L-α-phosphatidylcholine also had a significant impact on fungal composition, with 34 and 37 fungal genera showing significant changes after treatment with L-α-phosphatidylcholine in the HF_VI and CF_VI groups, respectively (Supplementary Table S3). Among these, the abundance of 25 fungal genera (14 of which appeared simultaneously with choline in the PubMed papers) was significantly higher in the HF_VI_Choline group than in the HF_VI group. In addition, the abundance of 10 fungal genera (five of which appeared simultaneously with choline in the PubMed papers) in the CF_VI_Choline group was significantly higher than that in the CF_VI group (Supplementary Table S3). Among these differential fungal genera, three fungal genera, *Metarhizium*, *Rhizopus*, and *Trichoderma*, were significantly increased after L-α-phosphatidylcholine addition in both the HF_VI_Choline and CF_VI_Choline groups.

**FIGURE 5:**
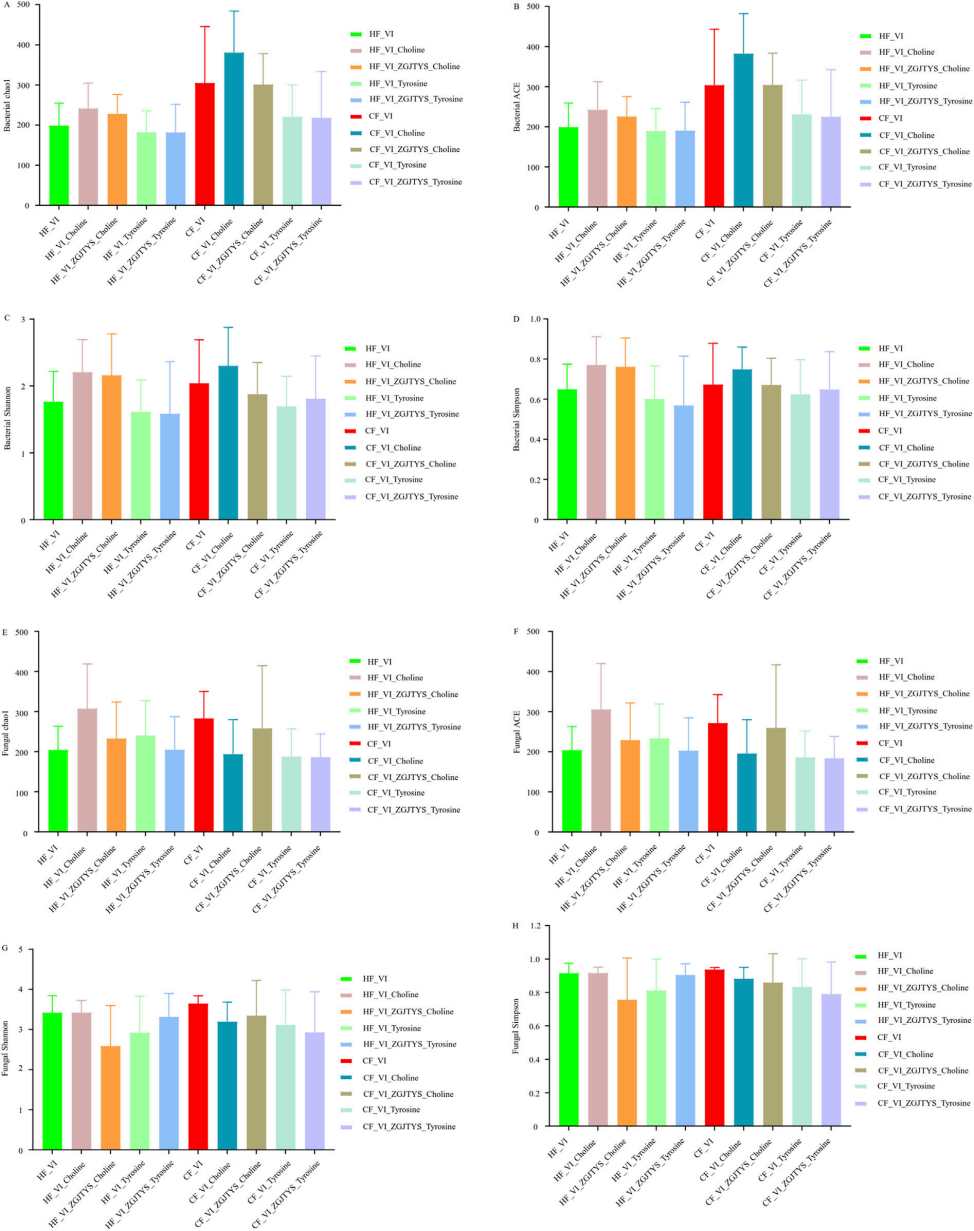
Comparative analysis of alpha diversity alterations following regulatory interventions of L-α-phosphatidylcholine, L-tyrosine and ZGJTYS. **(A−D)** show the bacterial alpha diversity results. **(E−H)** show the fungal alpha diversity results.

Given the established clinical efficacy of ZGJTYS in DKD management, we specifically investigated its regulatory effects on gut microbiota associated with L-α-phosphatidylcholine. Figure 5 demonstrates that L-α-phosphatidylcholine administration elevated bacterial and fungal alpha diversity indices in the HF_VI group and bacterial diversity in the CF_VI group while reducing fungal diversity in the CF_VI group. Notably, ZGJTYS co-administration restored alpha diversity parameters across all experimental groups to baseline levels observed without L-α-phosphatidylcholine intervention, except for persistent alterations in fungal Shannon and Simpson indices within the HF_VI_ZGJTYS_Choline group. Lefse analysis further demonstrated that four bacterial genera (which has been found to co-occur with choline in the PubMed literature) and 22 fungal genera (including 13 genera that has also been associated with choline in PubMed publications) no longer exhibited significant differences between the HF_VI and HF_VI-ZGJTYS_Choline groups (Supplementary Figure S7; Tables 2, 3). These organisms had originally exhibited significant differences between the HF_VI and HF_VI_Choline groups (Tables 2, 3). Similarly, 24 significantly different bacterial genera (that appeared simultaneously with choline in the PubMed paper), and eight significantly different fungal genera (including three fungal genera that appeared simultaneously with L-α-phosphatidylcholine in the PubMed paper) no longer exhibited significant differences between CF_VI and CF_VI_ZGJTYS_Choline (Supplementary Figure S7; Tables 2, 3).

**TABLE 2:** Significantly changed bacterial abundance due to the addition of L-α-phosphatidylcholine.

Coexist in the literature^a^	Restored by ZGJTYS^a^	HF group	CF group
Y	Y	*Achromobacter*; *Barnesiella*; *Comamonas*; *Ralstonia*	*Acetobacter*; *Achromobacter*; *Acidovorax*; *Brevundimonas*; *Corynebacterium*_1; *Delftia*; *Erysipelothrix*; *Flavobacterium*; *Lachnospiraceae*_NK3A20_group; *Micrococcus*; *Nocardioides*; *Paracoccus*; *Pedobacter*; *Prevotella*_7; *Prevotella*_9; *Prevotellaceae*_NK3B31_group; *Pseudomonas*; *Rhizobium*; *Rhodobacter*; *Rickettsia*; *Staphylococcus*; *Sphingomonas*; *Rikenellaceae*_RC9_gut_group; *Stenotrophomonas*
	N	*Alistipes*; *Anaerotruncus*; *Bacteroides*; *Butyricimonas*; *Faecalibacterium*; *Flavonifractor*; *Haemophilus*; *Halomonas*; *Odoribacter*; *Oscillospira*; *Parabacteroides*; *Pseudomonas*; *Roseburia*; *Microcella*; *Ruminococcaceae*_NK4A214; *Paraprevotella*; *Ruminococcaceae*_UC003; *Serratia*; *Phascolarctobacterium*; *Sutterella*; *Eubacterium_rectale*	*Acinetobacter*; *Aeromonas*; *Alistipes*; *Bacteroides*; *Comamonas*; *Halomonas*;*Lactobacillus*; *Paenibacillus*; *Parabacteroides*; *Propionibacterium; Ralstonia*; *Serratia*; *Cetobacterium*; *Chryseobacterium*; *Microcella*; *Clostridium_sensu_stricto*_12; *Glutamicibacter*; *Pseudobutyrivibrio*
N	N	*Eubacterium_ventriosum*; *Aliihoeflea*; *Pelagibacterium*; *Sphingobacterium*; *Ruminiclostridium*_9	*Aliihoeflea*; *Alishewanella*; *Aquamicrobium*; *Gilliamella*; *Myroides*; *Nesterenkonia; Pandoraea*; *Pelagibacterium*; *Peredibacter*; *Pusillimonas*; *Sphingobacterium*; *Wolbachia*

^a^ , Y represents yes, N represents no.

**TABLE 3:** Significantly changed fungi due to the addition of L-α-phosphatidylcholine, L-tyrosine and ZGJTYS.

Type of fungi	Literature relevance	Restored by ZGJTYS	Changed fungi in group HF	Changed fungi in group CF
Choline regulated	Coexist with choline in the literature	Y	*Acremonium*; *Cercospora*; *Enterocarpus*; *Fusarium*; *Ganoderma*; *Glycine*; *Penicillium*; *Phoma*; *Pichia*; *Rhizopus*; *Scopulariopsis*; *Talaromyces*; *Trichoderma*	*Metarhizium*; *Mucor*; *Trichoderma*
		N	*Metarhizium*	*Clonostachys*; *Rhizopus*
	Not coexist with choline in the literature	Y	*Ascobolus; Graphium*; *Microascus*; *Paraconiothyrium*; *Phanerodontia*; *Phyllosticta*; *Pseudogymnoascus*; *Roussoella*; *Xerochrysium*	*Filobasidium*; *Leiotrametes*; *Naganishia*; *Sporobolomyces*; *Wallemia*
		N	*Prototheca*; *Xeromyces*	
Tyrosine regulated	Coexist with tyrosine in the literature	Y	*Epicoccum*; *Metarhizium*; *Microascus*; *Millerozyma*; *Rhizopus*	*Alternaria*; *Candida*; *Metarhizium*; *Oryza*; *Prunus*; *Rhizopus*; *Rhodotorula*; *Saccharomyces*; *Ustilaginoidea*
		N	*Penicillium*; *Prototheca*; *Talaromyces*	
	Not coexist with tyrosine in the literature	Y	*Xerochrysium*	*Kurtzmaniella*
		N	*Xeromyces*	

Note: Y represents these fungi were restored by adding ZGJTYS; N represents these fungi cannot be restored by adding ZGJTYS.

### 3.5 Regulatory effect of ZGJTYS on the intestinal flora response to L-tyrosine

In an analysis of the interventional functions of L-tyrosine, the alpha diversity results showed that the L-tyrosine intervention had little effect on the bacterial and fungal diversity in the HF_VI group (Figure 5; Supplementary Table S2). The ACE, Chao1, Shannon, Observed_species, Shannon, and Simpson indexes did not show significant changes (Supplementary Table S2). However, L-tyrosine had a significant impact on the fungal diversity in the CF_VI group. The ACE, Chao1, and Observed_species indexes showed significant differences between the CF_VI and CF_VI_Tyrosine groups (Supplementary Table S2). The beta diversity analysis also showed that the L-tyrosine addition did not significantly change the bacterial composition of HF_VI and CF_VI. The HF_VI group had a relatively close relationship with the HF_VI_Tyrosine group and were clustered in the same branch, while the CF_VI group had a relatively close relationship with the CF_VI_Tyrosine group and were clustered in another branch (Figure 4E). The effects of L-tyrosine on the fungal composition in the HF_VI group was not significant. However, the CF_VI_Tyrosine group moved to the right as a whole and clustered separately in another branch after adding L-tyrosine (Figure 4F). The Lefse analysis also revealed that there was no significant difference in bacteria between the HF_VI and HF_VI_Tyrosine groups. Only four bacterial genera (*Ruminococcus_1*, *Eubacterium__ventriosum_group*, *Aliihoeflea*, *Flavonifractor)* were significantly higher in the CF_VI group than in the CF_VI_Tyrosine group (Supplementary Figure S8A). However, L-tyrosine has a relatively significant regulatory effect on fungi. In the HF group, significant differences were observed in the abundance of 32 fungal genera after the L-tyrosine addition (HF_VI_Tyrosine) (Supplementary Figure S8B). Among these, 10 fungal genera (eight of which appeared simultaneously with tyrosine in the papers) with significantly higher abundance in the HF_VI_Tyrosine group than that in the HF_VI group. After the L-tyrosine addition, the abundance of 29 fungal genera in the CF_VI group was significantly higher than that in the CF_VI_Tyrosine group (Supplementary Figure S8C), and another 10 fungal genera (nine of which appeared simultaneously with tyrosine in the PubMed paper) were significantly higher in the CF_VI_Tyrosine group. In both the HF_VI and CF_VI groups, the relative abundance of *Metarhizium* and *Rhizopus* significantly increased, and the relative abundance of *Apiotrichum*, *Cutaneotrichosporon*, *Diutina*, *Galactomyces*, *Geotrichum*, *Kodamaea*, *Saprochaete*, and *Trichosporon* significantly decreased after the addition of L-tyrosine.

A further analysis was performed on the effects of ZGJTYS and L-tyrosine co-treatment on the gut microbiota. While alpha diversity analysis indicated comparable profiles between the co-treatment and tyrosine-only supplementation (Figure 5; Supplementary Table S2), Lefse analysis identified several differences in the fungal community: six genera in the HF_VI_ZGJTYS_Tyrosine vs. HF_VI comparison and ten genera in the CF_VI_ZGJTYS_Tyrosine vs. CF_VI comparison no longer showed statistically significant differences (Supplementary Figure S9; Table 3). These microbial taxa had previously demonstrated significant differences between tyrosine-supplemented taxa and their non-supplemented counterparts.

### 3.6 Analysis of the metabolism difference between healthy volunteers and DKD patients after adding L-α-phosphatidylcholine and L-tyrosine

To determine the metabolic differences caused by the addition of L-α-phosphatidylcholine and L-tyrosine, a non-target metabolomics analysis was used to identify the different metabolites between the HF_VI and CF_VI groups. In all, 144 compounds were significantly higher in the CF_VI group, and 48 compounds were significantly higher in the HF_VI group, which resulted in significant enrichment of 36 Kyoto Encyclopedia of Genes and Genomes (KEGG) metabolic pathways (Supplementary Table S4). The top five KEGG metabolic pathways with significant impact were linoleic acid metabolism, flavonoid degradation, ABC transporters, the phosphatotransferase system (PTS), and cocaine addiction.

A further analysis was conducted on the L-α-phosphatidylcholine and L-tyrosine effects on HF and CF metabolites. The results showed that 38 compounds were significantly upregulated and 21 compounds were significantly downregulated in the HF_VI_Choline group compared to the HF_VI group, which resulted in the significant enrichment of seven KEGG metabolic pathways (Supplementary Table S4). A total of 33 compounds were detected as significantly upregulated and 43 compounds were detected as significantly downregulated in the CF_VI_Choline group compared to the CF_VI group, which resulted in significant enrichment of 17 KEGG metabolic pathways (Supplementary Table S4). A total of 35 compounds were significantly upregulated and 30 compounds were significantly downregulated in the HF_VI_Tyrosine group compared to the HF_VI group, which resulted in significant enrichment of 17 KEGG metabolic pathways (Supplementary Table S4). A total of 19 compounds were detected to be significantly upregulated and 34 compounds were detected to be significantly downregulated in the CF_VI_Tyrosine group compared to the CF_VI group that resulted in significant enrichment of 10 KEGG metabolic pathways (Supplementary Table S4).

**FIGURE 6:**
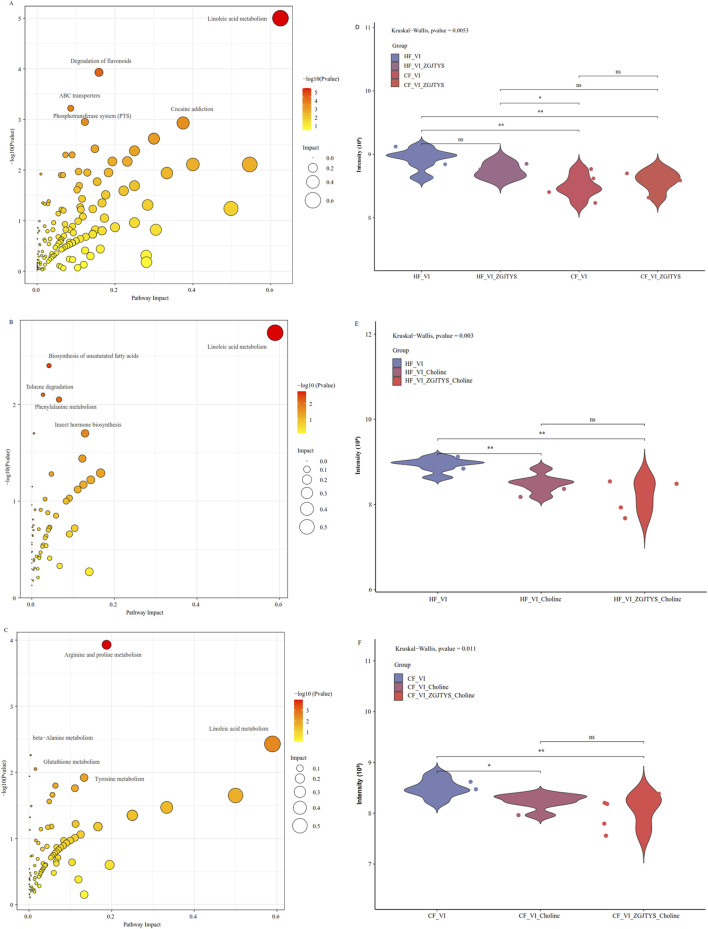
Bubble plot of the enriched KEGG pathways and content distribution of linoleic acid. **(A−C)** are bubble plots of the enriched KEGG pathways, while **(D−F)** are comparison plots of the linoleic acid contents. The X-axis in the bubble plot represents the degree of the KEGG enrichment of substances, the Y-axis represents enriched KEGG pathways, the color on the right indicates the P-value (Pvalue) of the hypergeometric distribution test for the KEGG enrichment, and the circle size represents the number of enriched substances. CF represents the DKD group, and HF represents the healthy college student volunteer control group. **(A)** HF_VI vs. CF_VI; **(B)** HF_VI vs. HF_VI_Choline; **(C)** CF_VI vs. CF_VI_Choline.

Among the enriched pathways, linoleic acid metabolism was significantly enriched between the HF_VI vs. CF_VI groups, the HF_VI vs. HF_VI_Choline groups, and the CF_VI vs. CF_VI_Choline groups (Figures 6A–C; Supplementary Figure S10A). A further analysis revealed that the linoleic acid content in the CF_VI group was significantly lower than that in the HF_VI group, and both groups showed a significant decrease in the linoleic acid content after L-α-phosphatidylcholine treatment (Figures 6D–F).

### 3.7 Analysis of the intervention effect of ZGJTYS on the metabolic regulation of L-α-phosphatidylcholine and L_tyrosine

**FIGURE 7:**
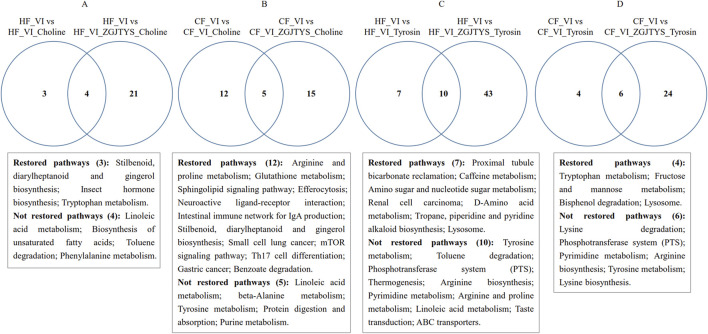
Venn diagram analysis showing significantly enriched KEGG pathways following supplementation with L-α-phosphatidylcholine, L-tyrosine, and ZGJTYS. **(A)** Number of significantly enriched KEGG pathways for the comparisons HF_VI vs HF_VI_Choline and HF_VI vs HF_VI_ZGJTYS_Choline. **(B)** Number of significantly enriched KEGG pathways for the comparisons CF_VI vs CF_VI_Choline and CF_VI vs CF_VI_ZGJTYS_Choline. **(C)** Number of significantly enriched KEGG pathways for the comparisons HF_VI vs HF_VI_Tyrosine and HF_VI vs HF_VI_ZGJTYS_Tyrosine. **(D)** Number of significantly enriched KEGG pathways for the comparisons CF_VI vs CF_VI_Tyrosine and CF_VI vs CF_VI_ZGJTYS_Tyrosine. Restored pathways indicates pathways that lost significant enrichment after adding ZGJTYS. Not restored pathways indicates pathways that retained significant enrichment after adding ZGJTYS.

When L-α-phosphatidylcholine was added alone, a total of 21 and 38 metabolites were identified as being significantly lower and higher in the HF_VI group than in the HF_VI_ Choline group, respectively. This resulted in a significant enrichment of 7 KEGG metabolic pathways. After co-treatment with L-α-phosphatidylcholine and ZGJTYS, 20 L-α-phosphatidylcholine-related metabolites (Supplementary Figure S11) and three KEGG pathways were restored; in other words, they no longer exhibited significant differences between the HF_VI vs. HF_VI_ZGJTYS_Choline groups (Figure 7). Between the CF_VI and CF_VI_Choline groups, 33 and 43 metabolites were identified as being significantly lower and higher in CF_VI group, respectively. This resulted in a significant enrichment of 17 KEGG metabolic pathways. After L-α-phosphatidylcholine and ZGJTYS intervention, 33 L-α-phosphatidylcholine related metabolites (Supplementary Figure S11) and 12 significantly enriched KEGG pathways were restored (Figure 7). These restored pathways consisted of efferocytosis, neuroactive ligand–receptor interaction, intestinal immune network for IgA production, small cell lung cancer, the mTOR signaling pathway, Th17 cell differentiation, and gastric cancer, which may be directly related to immunity and inflammation.

To further investigate the regulatory effect of the ZGJTYS intervention on metabolites after L_tyrosine addition, a total of 35 and 30 metabolites were found to be significantly lower and higher in the HF_VI group than in the HF_VI_Tyrosine group, respectively. This resulted in a significant enrichment of 17 KEGG metabolic pathways. After the L-tyrosine and ZGJTYS simultaneous intervention, 26 L-tyrosine related metabolites (Supplementary Figure S11) and seven significantly enriched KEGG pathways were restored (Figure 7). Between the CF_VI and CF_VI_Tyrosine groups, a total of 19 and 34 metabolites that were significantly lower and higher in CF_VI_Tyrosine group, respectively. This resulted in a significant enrichment of 10 KEGG metabolic pathways. After the simultaneous intervention with L-tyrosine and ZGJTYS, 13 tyrosine related metabolites (Supplementary Figure S11) and four significantly enriched KEGG pathways were restored (Figure 7).

## 4 Discussion

DKD remains the leading cause of ESRD worldwide. Our preliminary clinical study found that ZGJTYS improved abnormal glucose and lipid metabolism and reduce oxidative stress injury, urinary microalbumin, and mild inflammation in DKD patients (Chen et al., 2014; Cheng et al., 2010). However, mechanism underlying the effects of ZGJTYS in modulating DKD progression warrants further elucidation. Emerging evidence implicates gut microbiota dysbiosis in the pathogenesis of DKD-specific pathologies, including glomerular sclerosis and renal tubule injury (Liu et al., 2025). Given the established role of gut microbiome modulation in traditional Chinese medicine-based interventions for DKD (Ni et al., 2025), we systematically investigated the regulatory effects of ZGJTYS on intestinal microbial composition in DKD patients using an *in vitro* model.

We initially performed a comparative analysis of bacterial community composition between the CF and HF groups. The alpha diversity index was significantly lower in the CF group than in the HF group. The results of beta diversity analysis also showed that the HF and CF samples clustered well by groups. The LEfSe analysis revealed that three bacterial genera that included *Parabacterioids* and *Hungatella* were significantly higher in the CF group than in the HF group. However, eight bacterial genera that included *Prevotella_9* and *Coprococcus_3* were significantly lower in the CF group (Figure 2C). This result was basically consistent with the results reported in the literature. A meta-analysis by Wu et al. documented *Hungatella* enrichment and *Coprococcus* depletion in DKD group (Wu et al., 2024a). Similarly, Hu et al. and Zhang et al. reported diminished *Prevotella_9* abundance in DKD patients (Zhang et al., 2023; Hu et al., 2025). Intriguingly, *Parabacteroides* exhibited the opposite trends; Wang et al. (2022a) and Wang et al. (2022b) observed an increase in its abundance following Sacubitril/Valsartan treatment in a murine model of DKD (Wang et al., 2022b). Notably, among the differential genera between the CF and HF groups *Prevotella*_9 exhibited the highest relative abundance. Furthermore, its abundance increased in the CF_VI_ZGJTYS group when compared with the CF_VI group. Meanwhile, a previous study found that *Prevotella_9* exerted a protective effect against diabetic polyneuropathy (Xu et al., 2024). Collectively, these findings suggest that ZGJTYS may help ameliorate DKD progression through the targeted modulation of gut microbiota, particularly via the restoration of beneficial taxa such as *Prevotella_9*.

Numerous studies have shown that higher levels of L-α-phosphatidylcholine and tyrosine in the diet may be associated with the occurrence and development of DKD (Kwon et al., 2023; Liu et al., 2024). In this study, we compared the regulation of L-α-phosphatidylcholine and L-tyrosine on the intestinal flora in the HF and CF groups *in vitro*. The results showed that L-α-phosphatidylcholine had a significant effect on the bacterial and fungal composition in both the CF and HF groups. The literature search revealed that many published articles found that these regulated bacterial genera coexist with L-α-phosphatidylcholine. *Rickettsia* has been reported to have phosphorylcholine metabolic activity (Renesto et al., 2003; Winkler et al., 1994), and *Ralstonia solanacearum* encodes a choline-related protein NiaP that has the function of transporting nicotinate (Jeanguenin et al., 2012). Compared with L-α-phosphatidylcholine, L-tyrosine had weaker regulatory effect on the gut microbiota. No significantly regulated bacterial genera were detected by adding L-tyrosine in the HF group, and only four bacterial genera showed significant changes in their relative abundance after the addition of L-tyrosine in the CF group. However, L-tyrosine had a greater impact on the intestinal fungi composition. Based on a review of the literature, it can be concluded that many L-tyrosine-regulated fungal genera coexist with tyrosine. Szewczyk et al. found that tyrosine production by *Metarhizium* was significantly higher after atrazine stimulation (Szewczyk et al., 2020). Golebiowski et al. reported that *Metarhizium* may use tyrosine as an exogenous carbon source (Golebiowski et al., 2021).

ZGJTYS may play a role in preventing and treating DKD by helping patients recover their gut microbiota. Therefore, we further analyzed its intervention effects on the regulation of L-α-phosphatidylcholine and L-tyrosine-related microbiota. The results showed that four bacterial genera and 22 fungal genera related to L-α-phosphatidylcholine in the HF group recovered after the addition of ZGJTYS (Tables 2, 3). In the CF group, 25 bacterial genera and eight fungal genera recovered after the addition of ZGJTYS. Notably, certain pathogenic genera, including *Staphylococcus*, *Erysipelothrix*, *Pseudomonas,* and *Mucor*, are increased after adding *L-α-phosphatidylcholine*. However, these elevated microbial levels reverted to baseline after co-treatment with *L-α-phosphatidylcholine* and ZGJTYS. The addition of ZGJTYS and L-tyrosine simultaneously resulted in the recovery of five and nine fungal genera that were significantly regulated by L-α-phosphatidylcholine in the HF and CF groups, respectively. In the CF_VI_Tyrosine group, the abundance of *Candida*, a well-known opportunistic pathogen, was significantly higher than in the CF_VI group. However, co-treatment with L-tyrosine and ZGJTYS restored *Candida* levels in the CF_VI_ ZGJTYS_Tyrosine group to those observed in the CF_VI group. These findings suggest that ZGJTYS may potentially alleviate DKD progression by suppressing opportunistic pathogen proliferation.

We also performed a non-target metabolomics analysis. The results showed that the linoleic acid content in the CF group was significantly lower than that in the HF group, and both the HF and CF groups showed significant decreases in the linoleic acid contents after the addition of L-α-phosphatidylcholine. Because the decrease in the linoleic acid concentration has been reported to be related to the occurrence and development of DKD, this may be one of the reasons that the excessive intake of L-α-phosphatidylcholine increases the risk of DKD. Sha et al. reported that linoleic acid metabolism was significantly inhibited during DKD pathogenesis and progression (Sha et al., 2020), while Wu et al. found that an increased concentration of linoleic acid was involved in the protective mechanism of *Persicaria capitate* against DKD (Wu et al., 2024b). The mechanism by which linoleic acid relieves DKD may be related to it being one of the major omega-6 (n-6) polyunsaturated fatty acids reported to potentially modulate systemic immune inflammation (Shan et al., 2025). However, ZGJTYS intervention did not significantly alter the linoleic acid content (Figures 6D–F). We further analyzed the ZGJTYS intervention effects on the levels of other metabolites regulated by L-α-phosphatidylcholine and found that 20 L-α-phosphatidylcholine related metabolites and three significantly enriched KEGG pathways were restored in the HF group. Meanwhile, 33 L-α-phosphatidylcholine related metabolites and 12 significantly enriched KEGG pathways, were restored in the CF group. Among these pathways, the macrophage efferocytosis and mTOR signaling pathways have been reported to be associated with DKD occurrence and development (Song et al., 2024). Several studies have suggested that impaired macrophage efferocytosis aggravates the inflammatory response (Song et al., 2024), and restoring efferocytosis can alleviate DKD and some other metabolic diseases (Zhang et al., 2025). Considering that *Pseudomonas aeruginosa* can profoundly inhibit the alveolar macrophage clearance of apoptotic cells (efferocytosis) via the direct effect of virulence factors (Mccaslin et al., 2015), the increase in *Pseudomonas* after adding L-α-phosphatidylcholine has the potential to impair efferocytosis and aggravate the inflammatory response. The decrease of *Pseudomonas* in the CF_VI_Choline group after adding ZGJTYS indicated that ZGJTYS could have the potential to prevent and treat the DKD by regulating bacterial abundance, thus restoring efferocytosis to alleviate the inflammatory response.

With the addition of L-tyrosine, 65 metabolites were regulated in the HF group that resulted in significant enrichment of 17 KEGG metabolic pathways. After adding of ZGJTYS, a total of 26 tyrosine related metabolites and seven significantly enriched KEGG pathways were restored. In the CF group, 53 metabolites were regulated, which resulted in the significant enrichment of 10 KEGG metabolic pathways. After intervention with ZGJTYS, 13 tyrosine-related metabolites and four significantly enriched KEGG pathways were restored. Some tyrosine-related pathways, such as tryptophan metabolism, which are known to affect the occurrence, development, and prevention of DKD (Fiaccadori et al., 2020), were restored by using ZGJTYS. These results indicated that ZGJTYS also had certain preventive and therapeutic effects on tyrosine related DKD.

Although intestinal derived metabolites, such as tyrosine, L-α-phosphatidylcholine, tryptophan, P-cresol, phenol, trimethylamine, and indole, are closely related to the occurrence and development of DKD (Fiaccadori et al., 2020), only four compounds, namely, tyrosine, choline, tryptophan, and indole, were identified in this study. Except for tyrosine, which was significantly higher in the sample with the ZGJTYS addition, there was no significant difference in the three other compounds between these groups (Supplementary Figure S12). HPLC was also used to detect the phenol in fermentation broth samples. However, no phenol was detected in these samples. This may be due to the low level of these metabolites limiting their identification. More specific and sensitive methods for targeted detection of these intestinal derived metabolites are needed.

Although it has been reported that some botanical drugs can alleviate DKD symptoms clinically, the mechanism still needs to be further clarified (Shen et al., 2024; Deng et al., 2024). Some researchers have studied the mechanism of botanical drugs in preventing and treating DKD from the perspective of the host inflammatory response and signaling pathways (Shen et al., 2024; Deng et al., 2024). This study used an *in vitro* fermentation system to avoid interference from host factors and investigated how botanical drugs help alleviate DKD by regulating the gut microbiota and its metabolites. These results are beneficial for a better understanding of the complex relationship among botanical drugs, gut microbiota, dietary risk factors, and DKD. However, there are some limitations to this study. First, the number of clinical samples was relatively small, and most DKD patients had already used hypoglycemic drugs in the early stage. This may have affected the composition of their gut microbiota. Second, there may have been some differences between the *in vitro* fermentation and real human data, and this could have affected the accuracy of the analysis results. In addition, current metabolomics analyses are unable to accurately identify and analyze metabolites with low levels or those that have not yet been included in databases. The next step is to collect more clinical samples and adopt more advanced methods for the detection of gut microbiota and metabolites. Such strategies will be beneficial to better identify the different microorganisms and their metabolites between DKD patients and healthy volunteers, as well as to analyze the prevention and treatment mechanisms of drugs such as ZGJTYS on DKD disease.

In summary, we found that the diversity of gut microbiota in DKD patients was significantly lower, with 22 bacterial genera and four fungal genera significantly different between DKD patients and normal volunteers. The ROC diagnostic curve analysis that used *Parabacterioids* and *Prevotella_9* showed an AUC readiness greater than 0.75. L-α-phosphatidylcholine and L-tyrosine were found to regulate the gut microbiota and their metabolites *in vitro*, and ZGJTYS could effectively restore these changes. Although ZGJTYS shows a regulatory effect on gut microbiota and metabolites, further research is needed to determine whether there is a direct correlation between the differences in these metabolites and changes in the microbiota, as well as whether these regulatory effects are the main reasons for its ability to treat and prevent DKD.

## Data Availability

The data have been deposited in the sequence read archive (SRA) of the National Center for Biotechnological Information (NCBI) as GenBank Accession Number PRJNA1190047.
