# Supplementary material for: The effect of Zuogui-Jiangtang-Yishen decoction on the intestinal flora’s response to L-α-phosphatidylcholine and L-tyrosine in patients with diabetic kidney disease: an in vitro study
Source: Front Pharmacol. 2025 Jun 11;16:1573514. doi: 10.3389/fphar.2025.1573514 (PMC12188454; doi:10.3389/fphar.2025.1573514)
Supplement: Supplementary file 1 [file DataSheet1.docx]

**Supplementary Table 1. Number of samples used for high-throughput sequencing and non-target metabolomics analysis**

| Sample names | No. of 16S V3-V4 sequencing | No. of fungial ITS sequencing | No. of non-target metabolomics analysis |
| --- | --- | --- | --- |
| CF_Fecal | 12 | 12 | 0 |
| CF_VI | 12 | 12 | 6 |
| CF_VI_Choline | 12 | 12 | 6 |
| CF_VI_Tyrosine | 12 | 12 | 6 |
| CF_VI_ZGJTYS | 12 | 12 | 6 |
| CF_VI_ZGJTYS_Choline | 12 | 12 | 6 |
| CF_VI_ZGJTYS_Tyrosine | 12 | 12 | 6 |
| HF_Fecal | 12 | 12 | 0 |
| HF_VI | 12 | 12 | 6 |
| HF_VI_Choline | 12 | 12 | 6 |
| HF_VI_Tyrosine | 12 | 12 | 6 |
| HF_VI_ZGJTYS | 12 | 12 | 6 |
| HF_VI_ZGJTYS_Choline | 12 | 12 | 6 |
| HF_VI_ZGJTYS_Tyrosin | 12 | 12 | 6 |
| YF_Fecal | 12 | 12 | 0 |
| YF_VI | 12 | 12 | 6 |
| YF_VI_Choline | 12 | 12 | 6 |
| YF_VI_Tyrosine | 12 | 12 | 6 |
| YF_VI_ZGJTYS | 12 | 12 | 6 |
| YF_VI_ZGJTYS_Choline | 12 | 12 | 6 |
| YF_VI_ZGJTYS_Tyrosine | 12 | 12 | 6 |
| Total | 252 | 252 | 108 |

**Supplementary Table 2. P-values of the alpha diversity indexes for bacteria and fungi.**

|  | Comparison | ACE | Chao1 | Observed_species | Shannon | Simpson |
| --- | --- | --- | --- | --- | --- | --- |
| Bacterial | CF vs HF | 0.0083 | 0.0083 | 0.026 | 0.44 | 0.93 |
|  | CF vs YF | 0.045 | 0.06 | 0.043 | 0.8 | 1 |
|  | HF vs YF | 0.59 | 0.59 | 0.47 | 0.84 | 0.76 |
|  | HF_VI vs HF_VI_ZGJTYS | 0.89 | 0.76 | 0.56 | 0.84 | 0.84 |
|  | CF_VI vs CF_VI_ZGJTYS | 0.76 | 0.84 | 0.93 | 0.98 | 1 |
|  | HF_VI vs HF_VI_Choline | 0.039 | 0.052 | 0.043 | 0.045 | 0.014 |
|  | CF_VI vs CF_VI_Choline | 0.1 | 0.1 | 0.038 | 0.41 | 0.51 |
|  | HF_VI vs HF_VI_Tyrosine | 0.98 | 0.67 | 0.79 | 0.48 | 0.76 |
|  | CF_VI vs CF_VI_Tyrosine | 0.27 | 0.18 | 0.089 | 0.1 | 0.38 |
| Fungi | CF vs HF | 0.8 | 0.84 | 0.71 | 0.052 | 0.089 |
|  | CF vs YF | 0.51 | 0.44 | 0.55 | 0.76 | 0.84 |
|  | HF vs YF | 0.71 | 0.67 | 0.8 | 0.14 | 0.068 |
|  | HF_VI vs HF_VI_ZGJTYS | 0.11 | 0.1 | 0.065 | 0.84 | 0.8 |
|  | CF_VI vs CF_VI_ZGJTYS | 0.033 | 0.01 | 0.078 | 0.00072 | 0.0001 |
|  | HF_VI vs HF_VI_Choline | 0.0036 | 0.0029 | 0.0002 | 0.93 | 0.55 |
|  | CF_VI vs CF_VI_Choline | 0.017 | 0.012 | 0.026 | 0.0068 | 0.0045 |
|  | HF_VI vs HF_VI_Tyrosine | 0.24 | 0.13 | 0.2 | 0.16 | 0.1 |
|  | CF_VI vs CF_VI_Tyrosine | 0.0056 | 0.0023 | 0.0066 | 0.51 | 0.27 |

**Supplementary Table 3. Significantly changed bacteria and fungi due to the addition of L-α-phosphatidylcholine identified by using Lefse.**

|  |  | Increased after adding choline | Decreased after adding choline |
| --- | --- | --- | --- |
| HF Group | Bacteria | *Achromobacter*; *Aliihoeflea*; *Alistipes*; *Anaerotruncus*; *Bacteroides*; *Barnesiella*; *Butyricimonas*; *Comamonas*; *Eubacterium rectale group*; *Eubacterium ventriosum group*; *Faecalibacterium*; *Flavonifractor*; *Haemophilus*; *Halomonas*; *Microcella*; *Odoribacter*; *Oscillospira*; *Parabacteroides*; *Paraprevotella*; *Pelagibacterium*; *Phascolarctobacterium*; *Pseudomonas*; *Ralstonia*; *Roseburia*; *Ruminiclostridium_9*; *Ruminococcaceae NK4A214 group*; *Ruminococcaceae UCG-003*; *Serratia*; *Sphingobacterium*; *Sutterella* | *Actinomyces*; *Anaerostipes*; *Erysipelatoclostridium*; *Erysipelotrichaceae UCG-003*; *Gemella*; *Granulicatella*; *Klebsiella*; *Parvimonas*; *Ruminococcus gauvreauii group*; *Streptococcus*; *Subdoligranulum* |
|  | Fungi | *Acremonium*; *Ascobolus*; *Cercospora*; *Enterocarpus*; *Fusarium*; *Ganoderma*; *Glycine*; *Graphium*; *Metarhizium*; *Microascus*; *Paraconiothyrium*; *Penicillium*; *Phanerodontia*; *Phoma*; *Phyllosticta*; *Pichia*; *Prototheca*; *Pseudogymnoascus*; *Rhizopus*; *Roussoella; Scopulariopsis*; *Talaromyces*; *Trichoderma*; *Xerochrysium*; *Xeromyces* | *Lichtheimia*; *Malassezia*; *Neoascochyta*; *Piromyces*; *Saccharomyces*; *Starmerella*; *Sterigmatomyces*; *Tahromyces*; *Wallemia* |
| CF Group | Bacteria | *Acetobacter*; *Achromobacter*; *Acidovorax*; *Acinetobacter; Aeromonas*; *Aliihoeflea*; *Alishewanella*; *Alistipes*; *Aquamicrobium*; *Bacteroides*; *Brevundimonas*; *Cetobacterium*; *Chryseobacterium*; *Clostridium sensu stricto 12*; *Comamonas*; *Corynebacterium_1*; *Delftia*; *Erysipelothrix*; *Flavobacterium*; *Gilliamella*; *Glutamicibacter*; *Halomonas*; *Lachnospiraceae NK3A20 group*; *Lactobacillus; Microcella*; *Micrococcus*; *Myroides*; *Nesterenkonia*; *Nocardioides*; *Paenibacillus*; *Pandoraea*; *Parabacteroides*; *Paracoccus*; *Pedobacter*; *Pelagibacterium*; *Peredibacter*; *Prevotella_7*; *Prevotella_9*; *Prevotellaceae NK3B31 group*; *Propionibacterium*; *Pseudobutyrivibrio*; *Pseudomonas*; *Pusillimonas*; *Ralstonia*; *Rhizobium*; *Rhodobacter*; *Rickettsia*; *Rikenellaceae RC9 gut group*; *Serratia*; *Sphingobacterium*; *Sphingomonas*; *Staphylococcus*; *Stenotrophomonas*; *Wolbachia* | *Actinomyces*; *Blautia*; *Corynebacterium*; *Eggerthella*; *Erysipelatoclostridium*; *Escherichia_Shigella*; *Gemella*; *Gordonibacter*; *Granulicatella*; *Hungatella*; *Malikia*; *Peptostreptococcus*; *Rothia*; *Streptococcus* |
|  | Fungi | *Metarhizium*; *Wallemia*; *Mucor*; *Trichoderma*; *Rhizopus*; *Filobasidium*; *Sporobolomyces*; *Naganishia*; *Clonostachys*; *Leiotrametes* | *Magnusiomyces*; *Arachnomyces*; *Pseudocercospora*; *Elsinoe*; *Zopfiella*; *Sebacina*; *Triangularia*; *Bensingtonia*; *Paecilomyces*; *Yarrowia*; *Epicoccum*; *Phoma*; *Kodamaea*; *Arthrobotrys*; *Arthopyrenia*; *Cystoagaricus*; *Galactomyces*; *Ectophoma*; *Talaromyces*; *Saprochaete*; *Candida*; *Pichia*; *Kazachstania*; *Diutina*; *Geotrichum* |

**Supplementary Table 4. KEGG pathway enrichment analysis mediated by significantly differential metabolites.**

| Comparation | Name of KEGG pathways |
| --- | --- |
| HF_VI vs  CF_VI | linoleic acid metabolism; degradation of flavonoids; ABC transporters; phosphotransferase system; cocaine addiction; amphetamine addiction; alcoholism; arginine biosynthesis; dopaminergic synapse; lysine degradation; biosynthesis of unsaturated fatty acids; tyrosine metabolism; cutin, suberine and wax biosynthesis; PPAR signaling pathway; alanine, aspartate and glutamate metabolism; protein digestion and absorption; ferroptosis; aminobenzoate degradation; bacterial chemotaxis; cysteine and methionine metabolism; beta-Alanine metabolism; phenylalanine metabolism; phenylalanine, tyrosine and tryptophan biosynthesis; glutamatergic synapse; central carbon metabolism in cancer; vitamin digestion and absorption; GABAergic synapse; long-term depression; tryptophan metabolism; Parkinson disease; galactose metabolism; plant hormone signal transduction; histidine metabolism; D-Amino acid metabolism; arginine and proline metabolism; vitamin B6 metabolism |
| HF_VI vs  HF_VI_Choline | linoleic acid metabolism; biosynthesis of unsaturated fatty acids; toluene degradation; phenylalanine metabolism; stilbenoid, diarylheptanoid and gingerol biosynthesis; insect hormone biosynthesis; tryptophan metabolism |
| CF_VI vs CF_VI_Choline | arginine and proline metabolism; linoleic acid metabolism; beta-Alanine metabolism; glutathione metabolism; tyrosine metabolism; sphingolipid signaling pathway; protein digestion and absorption; efferocytosis; neuroactive ligand-receptor interaction; intestinal immune network for IgA production; purine metabolism; stilbenoid, diarylheptanoid and gingerol biosynthesis; small cell lung cancer; mTOR signaling pathway; Th17 cell differentiation; gastric cancer; benzoate degradation |
| HF_VI vs HF_VI_Tyrosin | tyrosine metabolism; toluene degradation; proximal tubule bicarbonate reclamation; phosphotransferase system; caffeine metabolism; thermogenesis; arginine biosynthesis; pyrimidine metabolism; amino sugar and nucleotide sugar metabolism; renal cell carcinoma; D-Amino acid metabolism; arginine and proline metabolism; linoleic acid metabolism; tropane, piperidine and pyridine alkaloid biosynthesis; lysosome; taste transduction; ABC transporters |
| CF_VI vs CF_VI_Tyrosin | tryptophan metabolism; fructose and mannose metabolism; lysine degradation; phosphotransferase system; pyrimidine metabolism; arginine biosynthesis; bisphenol degradation; tyrosine metabolism; lysine biosynthesis; lysosome |

**Supplementary Figure 1. Composition and relative abundance of bacteria and fungi in the original fecal samples.** A is the abundance chart of the bacterial genera, and B is the abundance chart of the fungal genera. CF represents the DN group, HF represents the healthy college student volunteer control group, and YF represents the middle-aged and elderly non-metabolic disease control group.

**
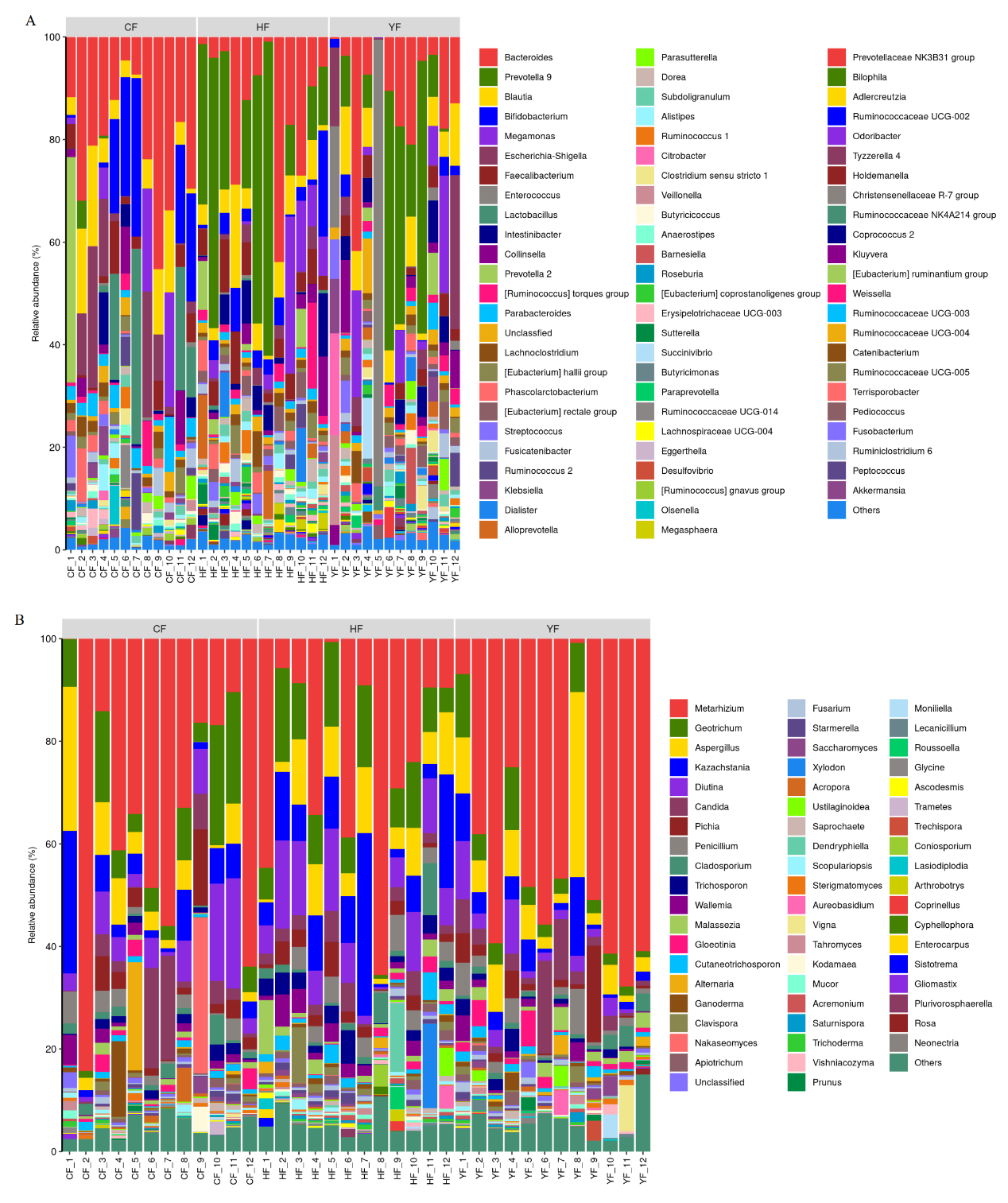
**

**Supplementary Figure 2. Lefse analysis of fungi in the original fecal samples.** CF represents the DN group, HF represents the healthy college student volunteer control group, and YF represents the middle-aged and elderly non-metabolic disease control group.
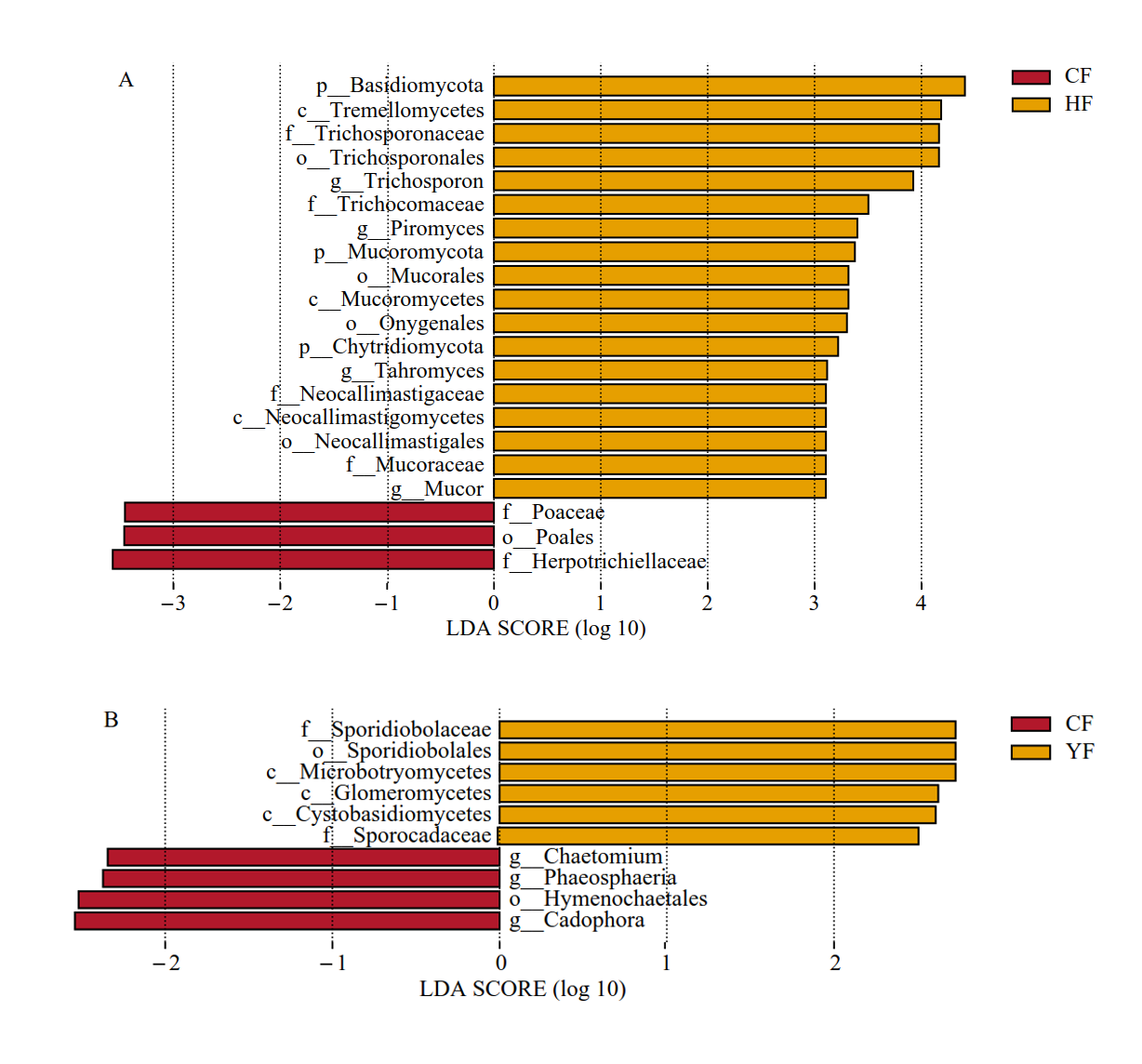


**Supplementary Figure 3. In vitro simulation effect of the VI media on fecal bacteria and fungi.** A is the results of the bacterial beta diversity analysis, and B is the results of the fungal beta diversity analysis.


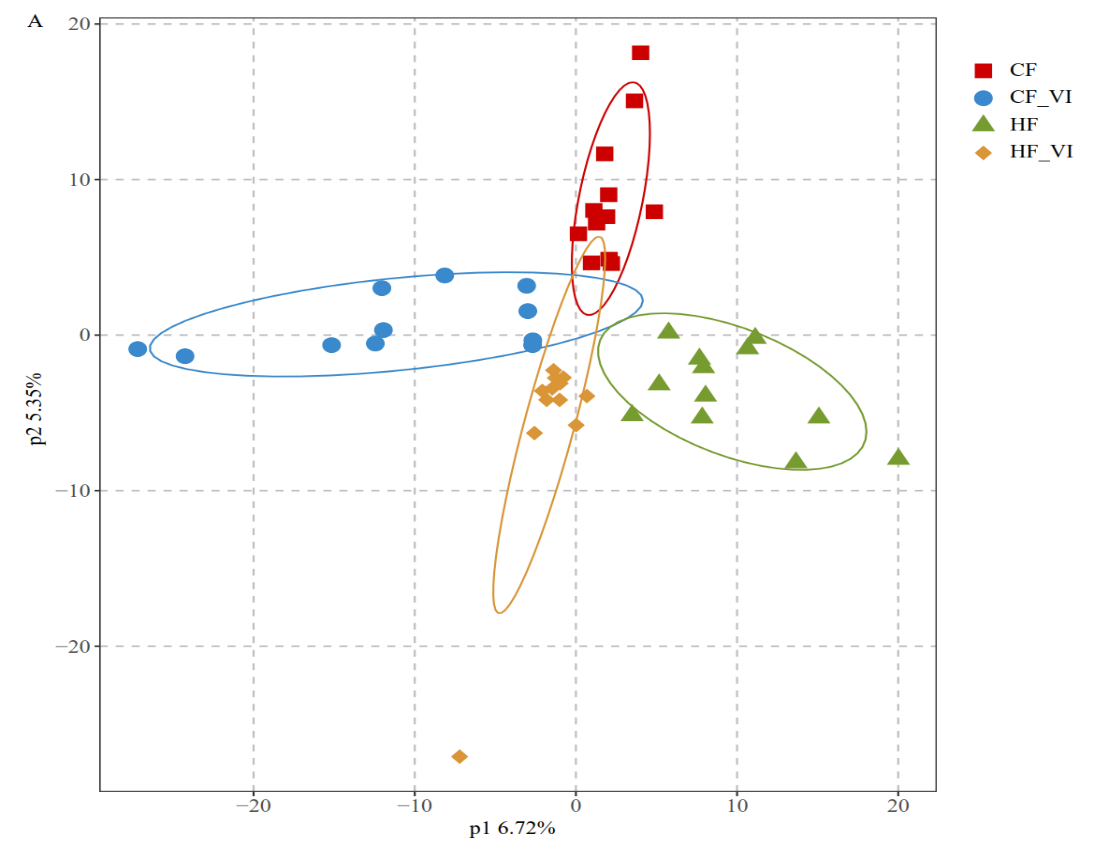


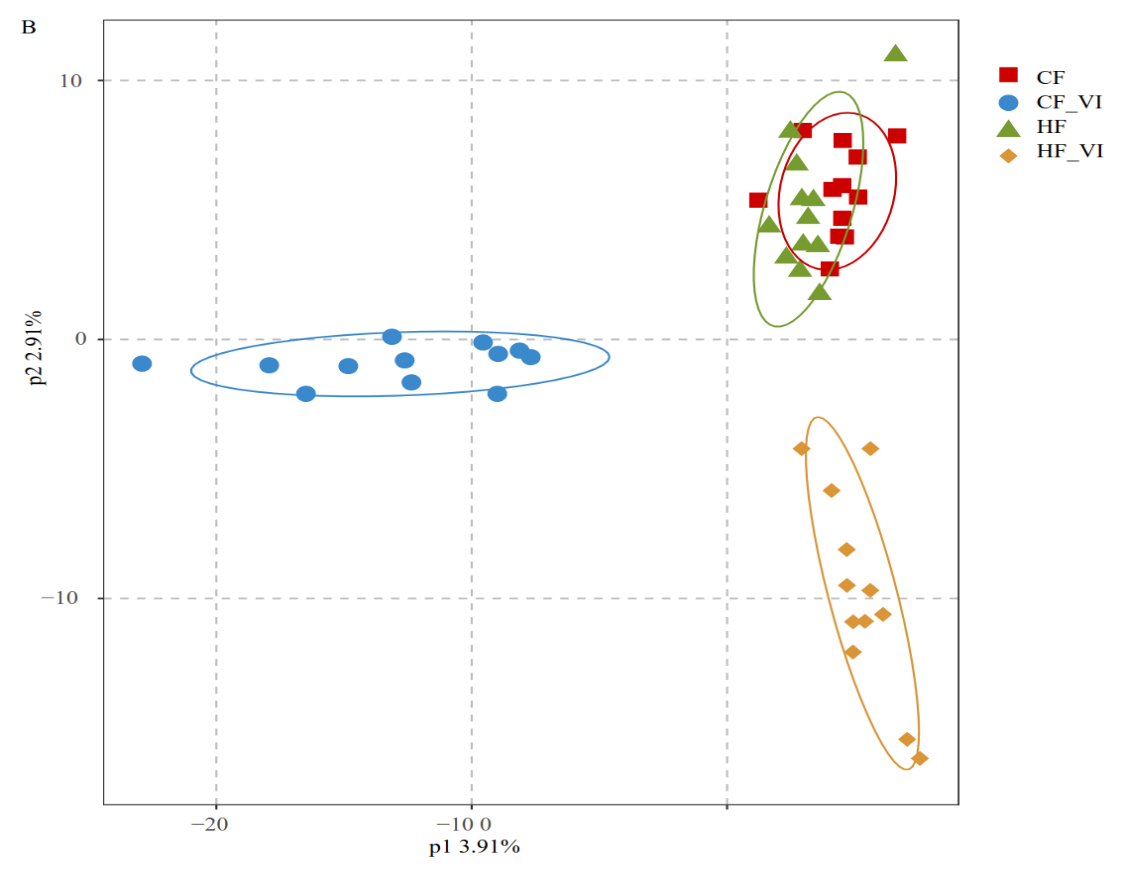


**Supplementary Figure 4. Lefse analysis of the significantly different bacterial in the fermented samples.** CF_VI represents the fecal bacteria obtained from the DN patients inoculated in the culture media *in vitro*. HF_VI represents the fecal bacteria obtained from healthy volunteers inoculated in the culture media *in vitro*.


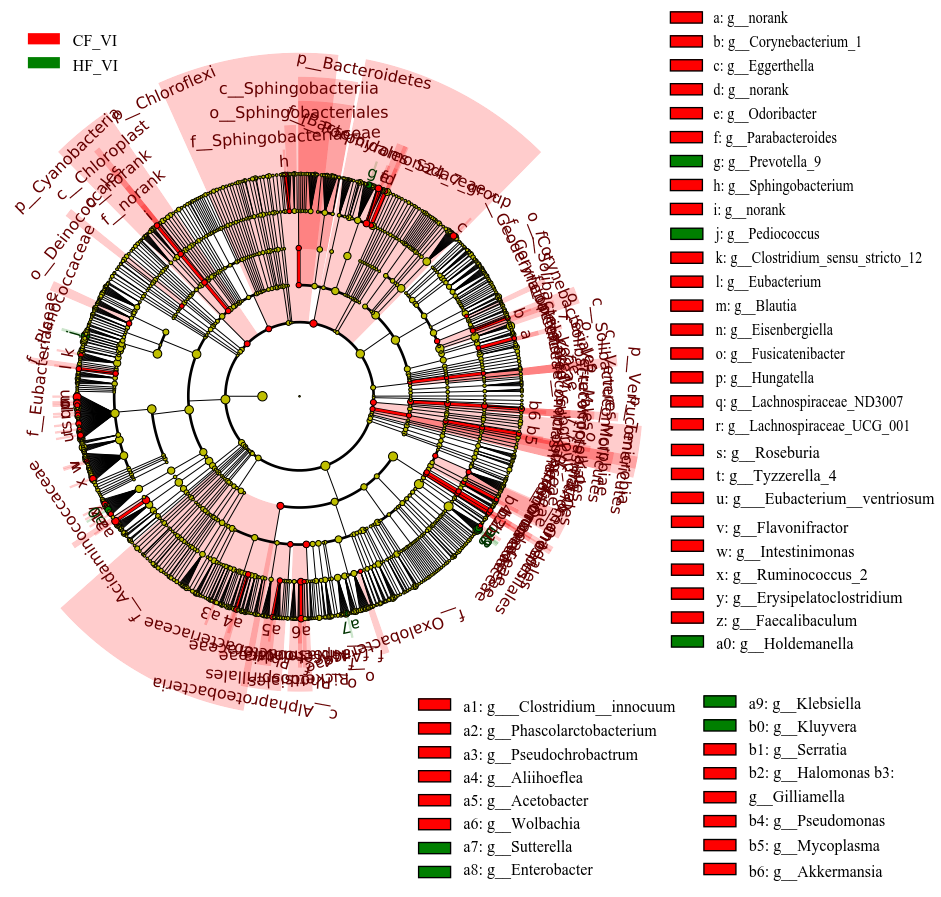


**Supplementary Figure 5. Enrichment analysis of the KEGG pathways before and after treatment with simulated upper gastrointestinal fluid.**

**
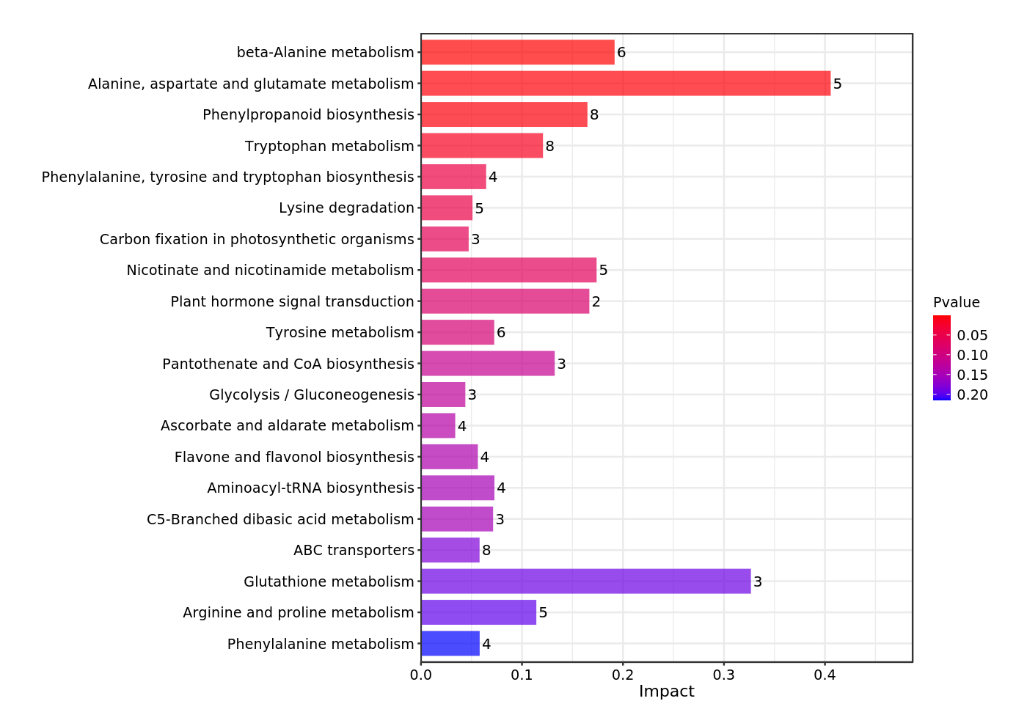
**

**Supplementary Figure 6. Lefse analysis of the effects of ZGJTYS on the bacterial and fungal composition.** A and B are the bacterial Lefse analysis results, and C and D are the fungal Lefse analysis results.


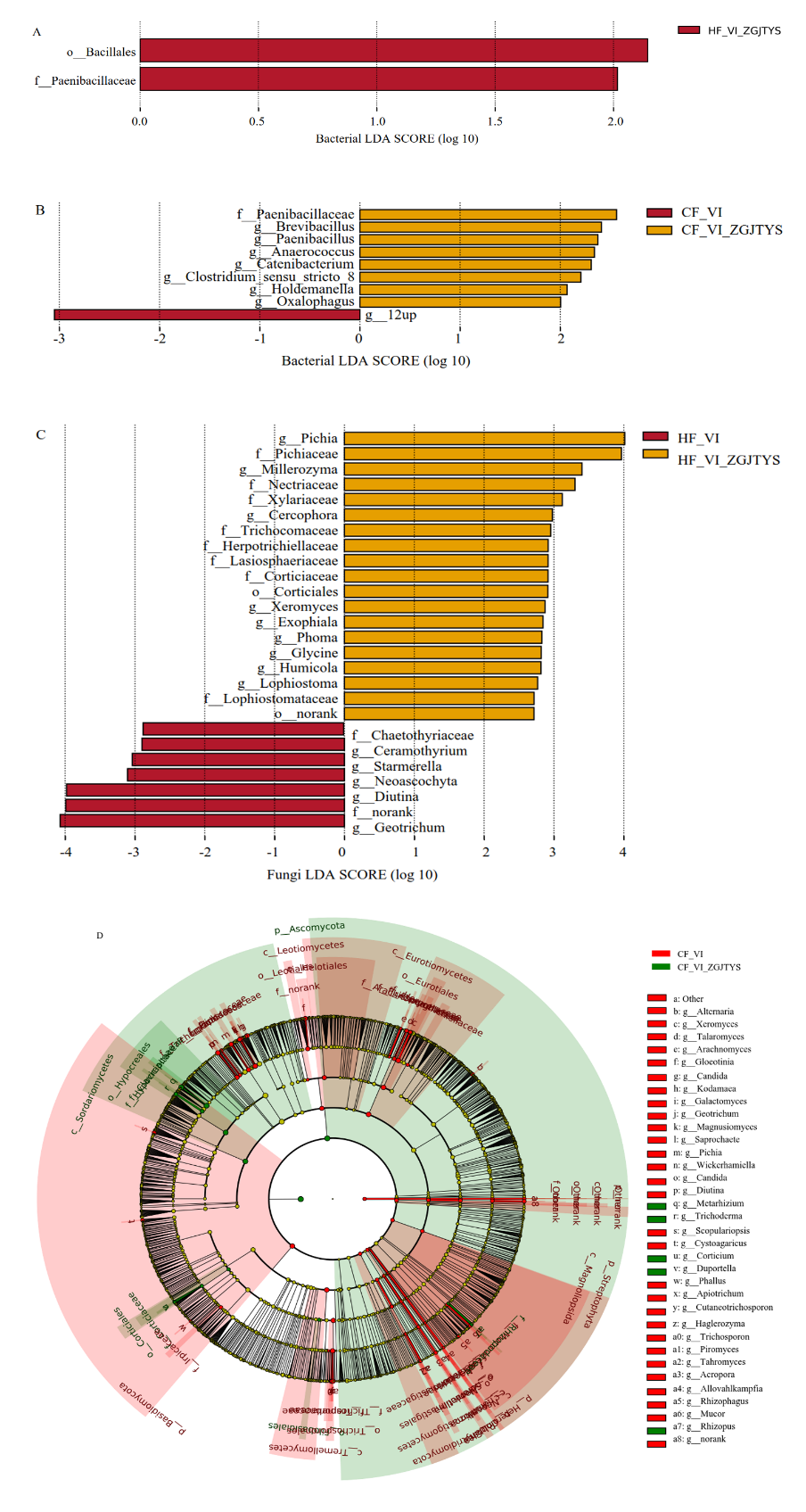


**Supplementary Figure 7. Lefse analysis of the effects of the simultaneous treatment with choline and ZGJTYS on the bacterial and fungal composition.** A and B are the bacterial Lefse analysis results. C and D are the fungal Lefse analysis results. CF represents the DN group, and HF represents the healthy college student volunteer control group.


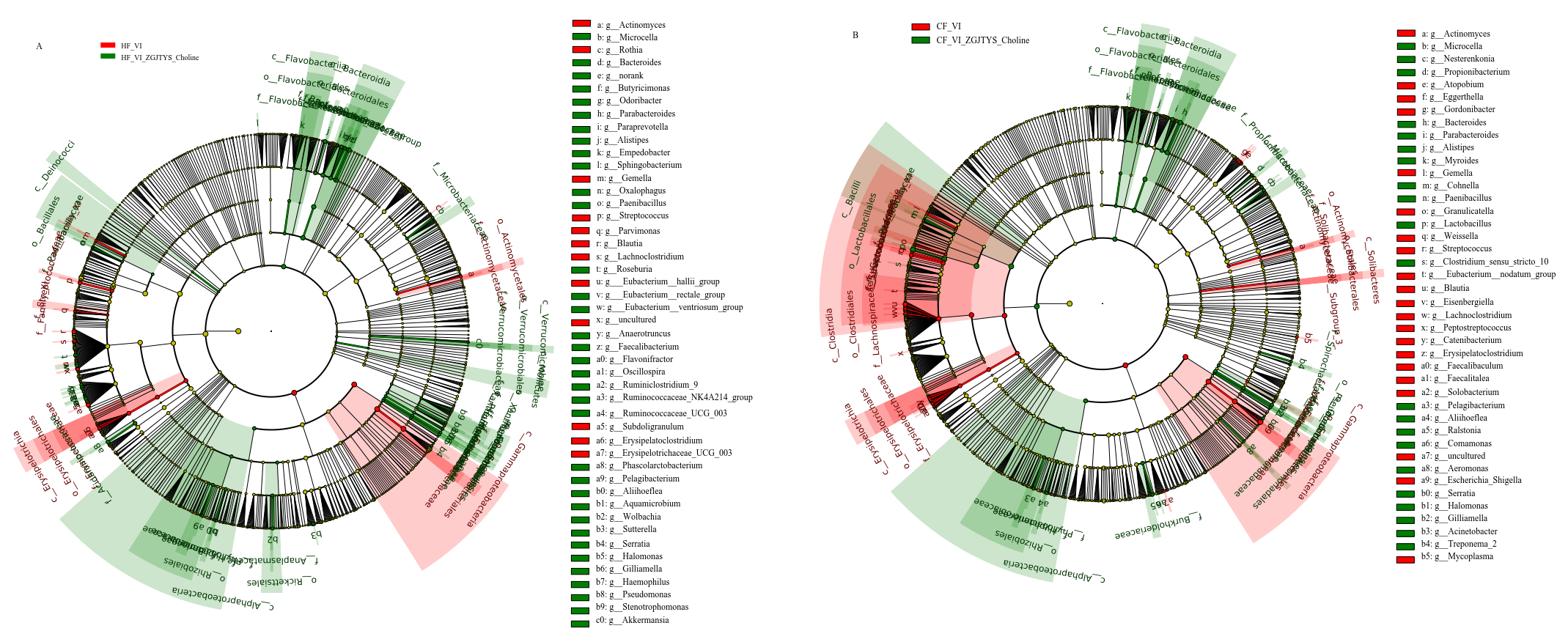


**
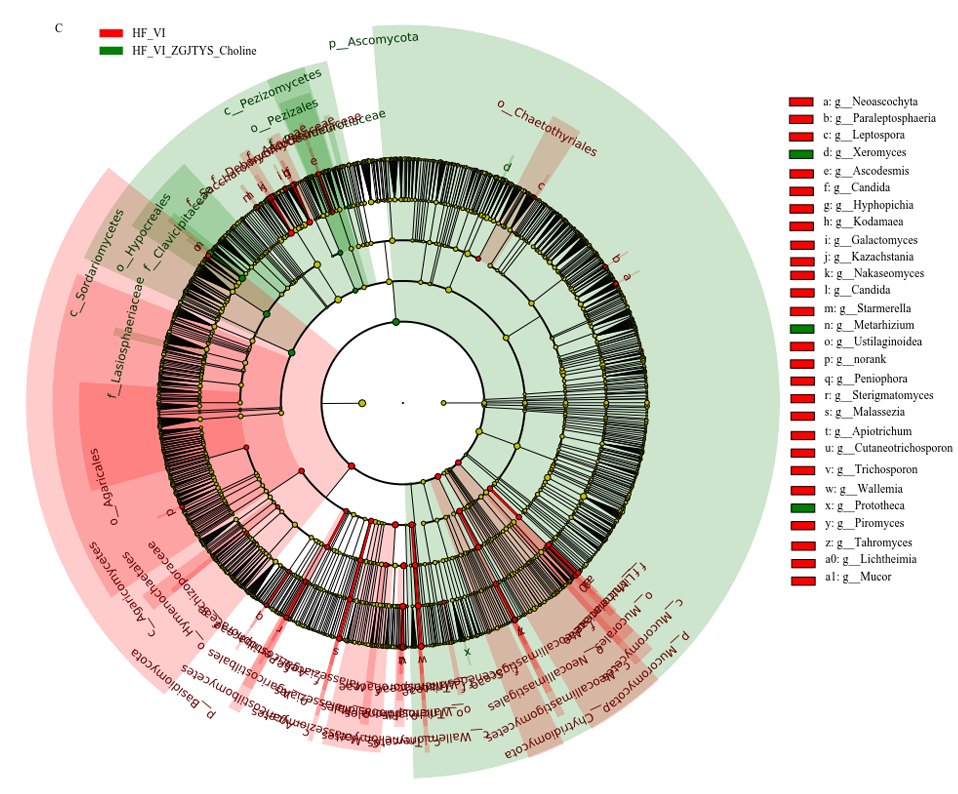

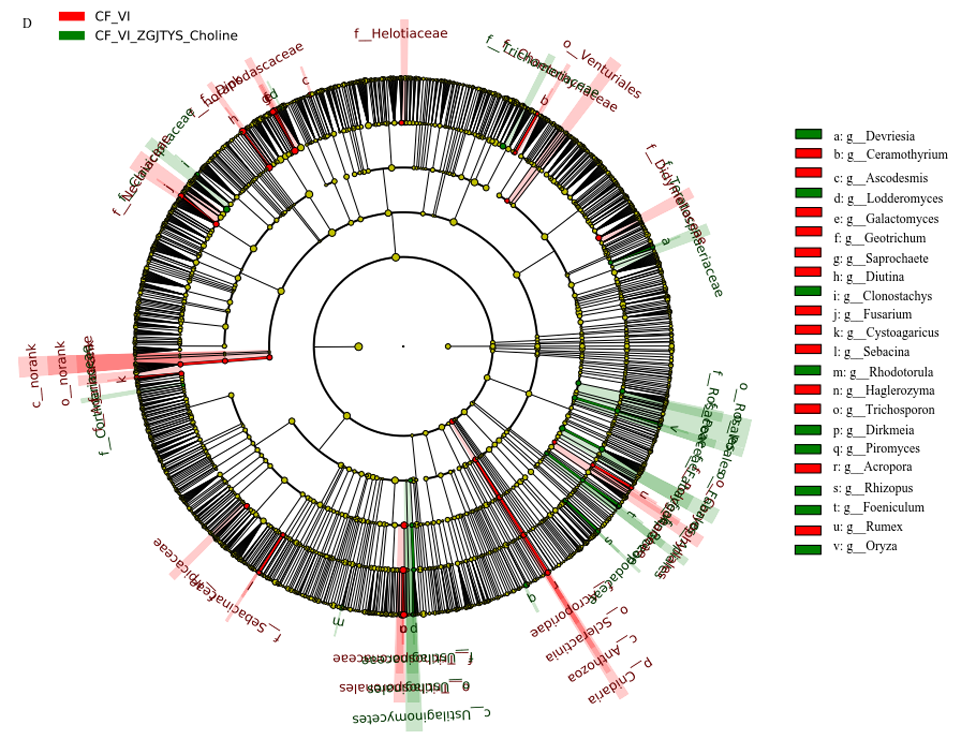
**

**Supplementary Figure 8. Lefse analysis of the effect of tyrosine on the bacterial and fungal composition.** A is the bacterial Lefse analysis result, B and C are the fungal Lefse analysis results.


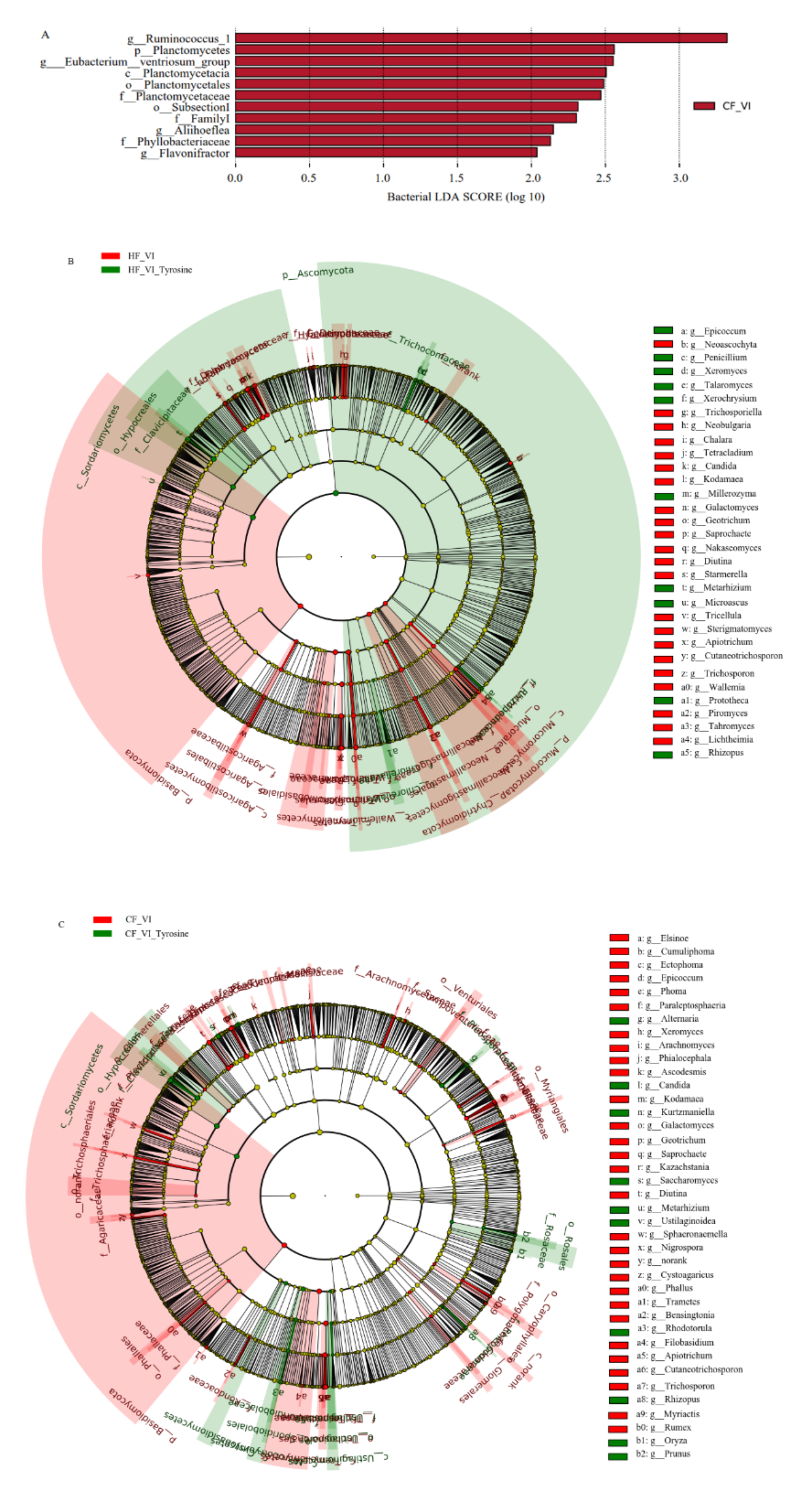


**Supplementary Figure 9. Lefse analysis of the combined effect of ZGJTYS and L-tyrosine on the fungal composition.** A is the bacterial Lefse analysis result, and B is the fungal Lefse analysis result.


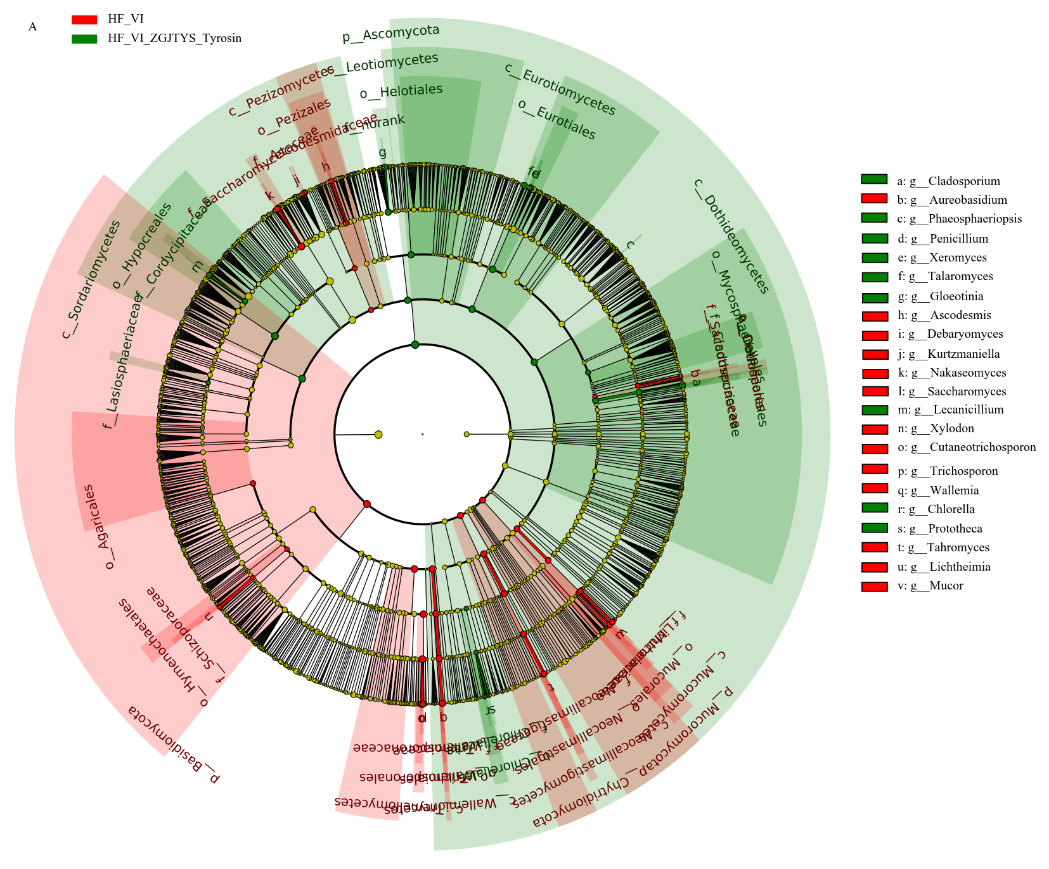

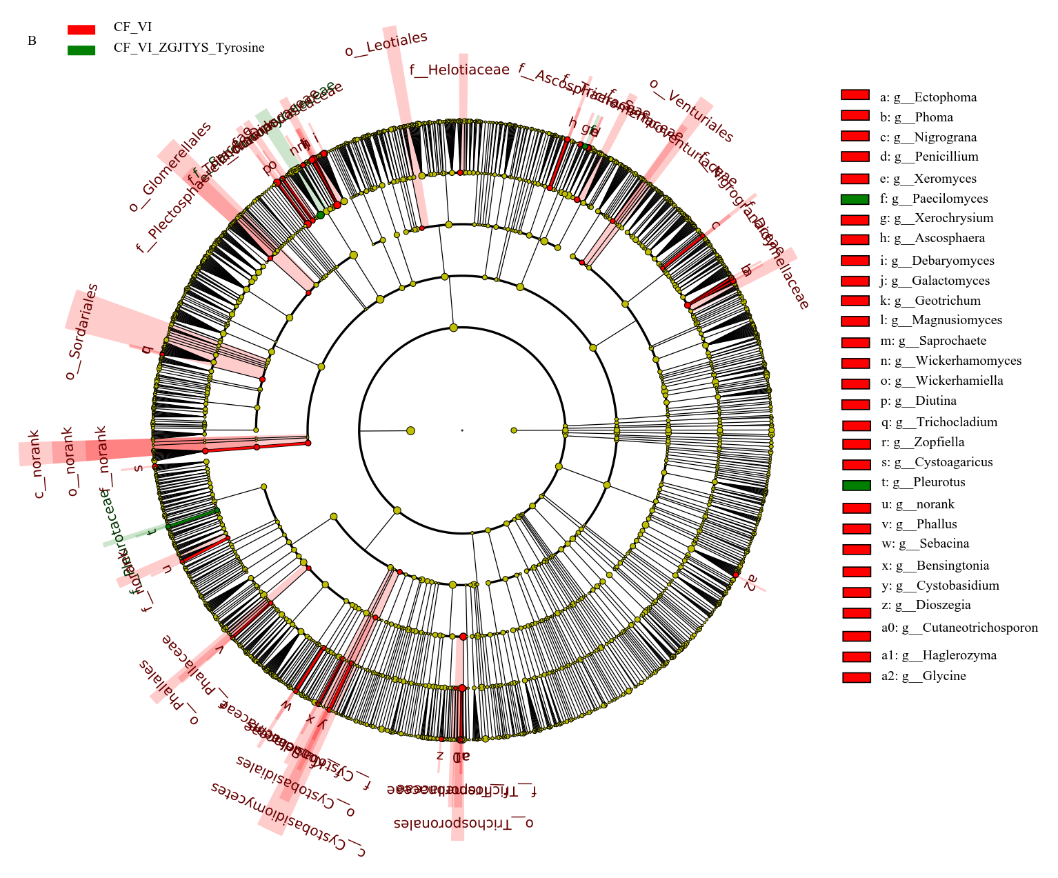


**Supplementary Figure 10. Venn plot analysis of the significant enriched KEGG pathways due to the choline and tyrosine treatment.** CF represents the DN group, and HF represents the healthy college student volunteer control group.


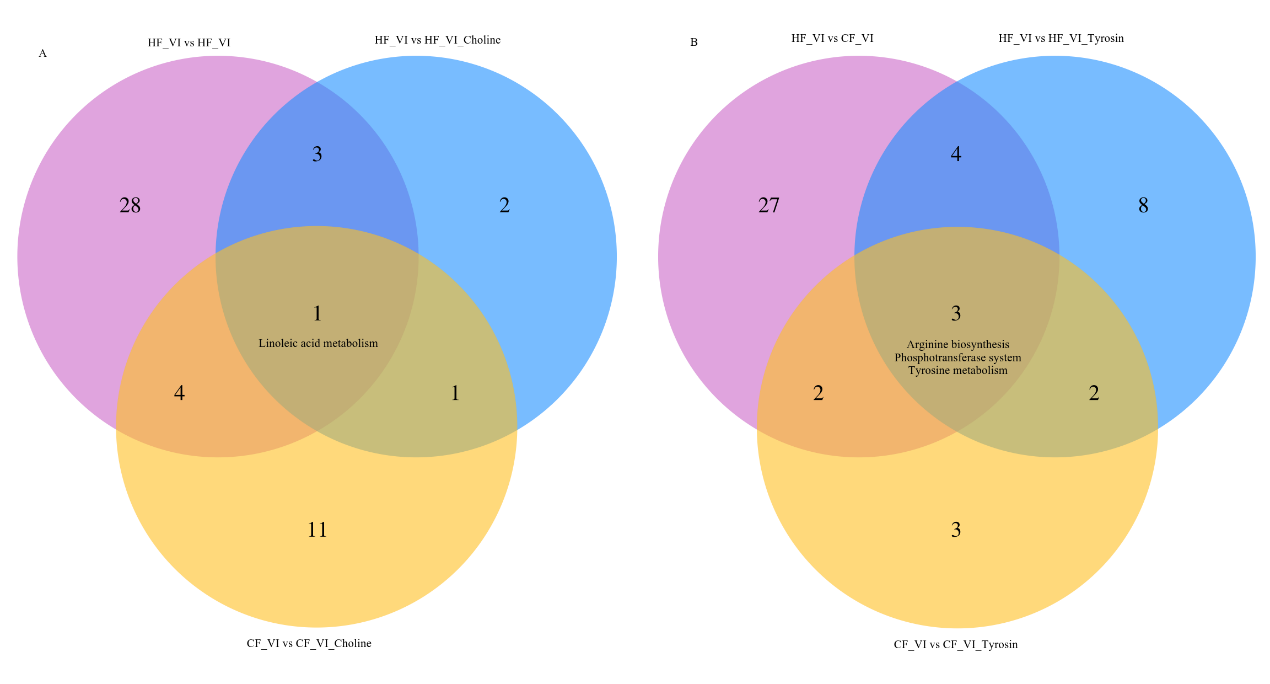


**Supplementary Figure 11. Venn plot analysis of the significant changed metabolites.**


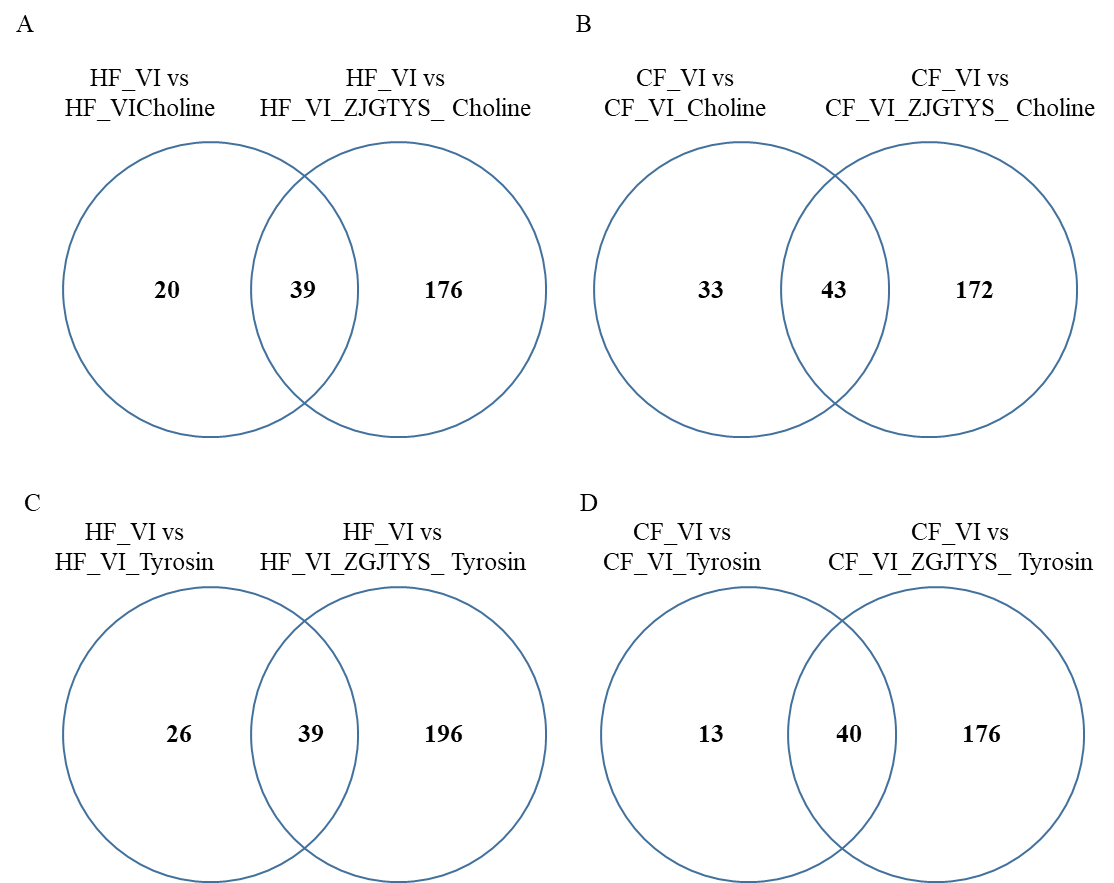


**Supplementary Figure 12. Content analysis of the four metabolites related to DN reported in the literature.**

**
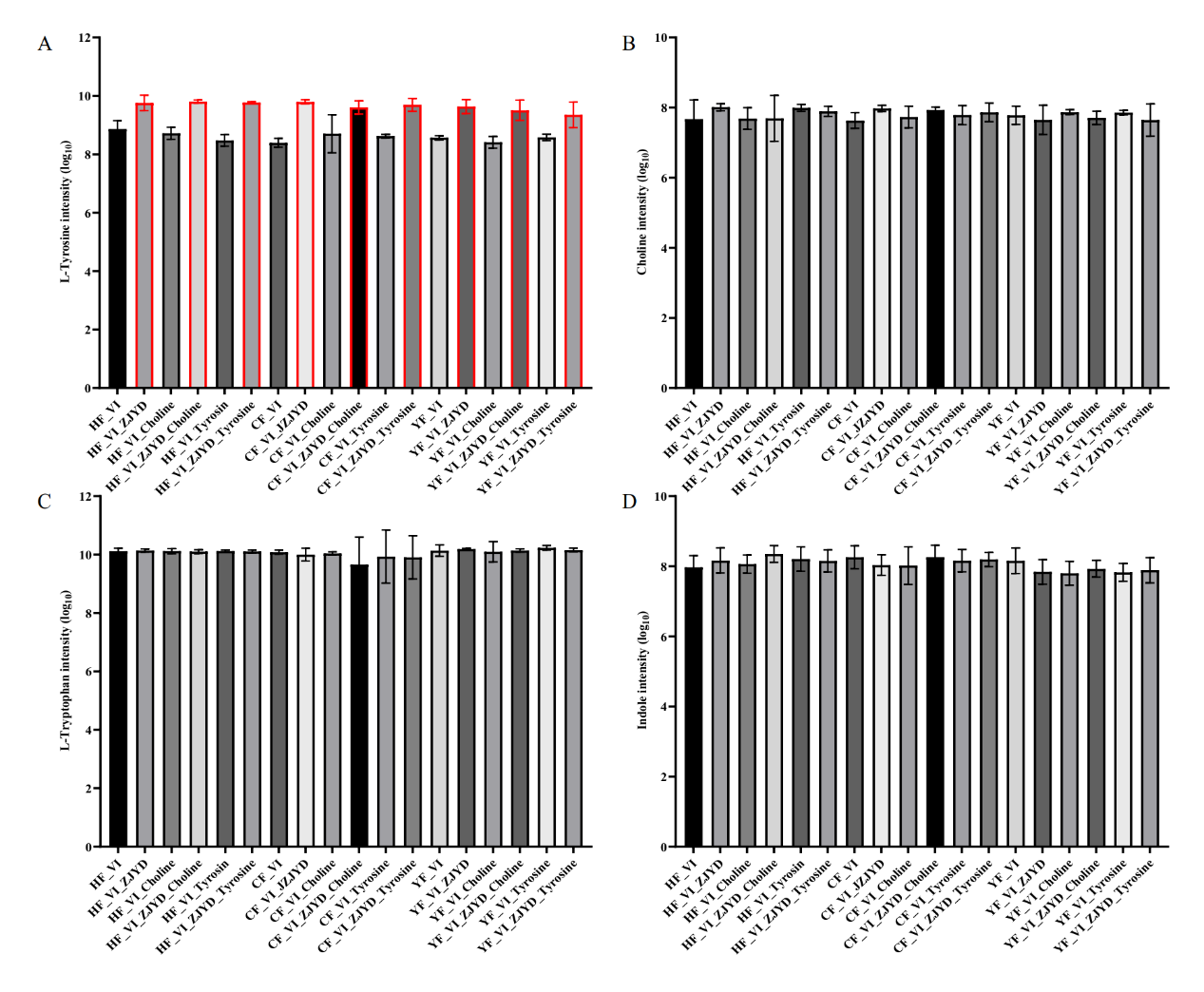
**
